# Ubiquitous and expanding glacier algal blooms modelled around the Greenland Ice Sheet

**DOI:** 10.1038/s43247-026-03758-8

**Published:** 2026-06-16

**Authors:** Christopher J. Williamson, Andrew J. Tedstone

**Affiliations:** 1 Bristol Glaciology Centre, University of Bristol, Bristol, UK; 2 Institute of Earth Surface Dynamics, University of Lausanne, Lausanne, Switzerland; 3 Department of Geosciences, University of Fribourg, Fribourg, Switzerland

**Keywords:** Ecological modelling, Water microbiology, Cryospheric science

## Abstract

Glacier algal blooms occupying the melting surface of the Greenland Ice Sheet (GrIS) support diverse microbial communities and amplify ice melt through positive albedo feedback. Knowledge about the distribution and magnitude of these blooms has been limited to isolated field surveys or regional remote sensing and modelling studies to-date. Here, we present Greenland-wide simulations of glacier algal blooms over the past two decades (2000–2022) using a Quasi Monte Carlo (QMC) ensemble approach informed through sensitivity analysis of key model parameters. We show how conditions conducive for glacier algal growth are present around the entirety of the GrIS margins each year of our study regardless of the magnitude and duration of the melt season. Spatiotemporal heterogeneity in accumulated biomass is modelled across the ablation zone, between ice sheet sectors and relative to inter-annual variability in melt. Bloom magnitude maps to the availability of ablation zone area in each year and we identify northern sectors as potential harbingers of expansive blooms that remain to be sampled. A small increasing trend in total bloom extent (3724 km^−2^ yr^−1^) between 2000 and 2022 supports the likelihood of future bloom expansion as more of the ice sheet is unlocked by warming.

## Abstract (web summary)

Glacier algal growth could occur around the entirety of the Greenland Ice Sheet margins regardless of the magnitude and duration of the melt season, suggests a Greenland-wide simulation of glacier algal blooms over the past two decades (2000–2022).

## Introduction

Streptophyte “glacier algae” proliferate in glacial surface ice when summer conditions provide the liquid water, nutrients and sunlight required for growth^1–7^. On the Greenland Ice Sheet (GrIS), widespread blooms initiate following snow line retreat^4,6,8^, with maximal cell densities recorded at 10^6^ cells mL^−1^ of melt water^1,4,6,9^. At these densities, glacier algae exacerbate surface melting by reducing the ice albedo, causing an additional 5.5–8.0 Gt of melt along the western ice sheet margin^10^. Given that GrIS mass loss represents the single largest cryospheric contributor to global eustatic sea level rise^11^, constraining the distribution and magnitude of Greenland’s glacier algal blooms remains a significant research priority^4,6,10,12^.

Advances in GrIS glacier algal bloom ecology have been achieved through a series of in situ field studies, remote sensing and regional modelling studies. Field surveys have confirmed glacier algal presence in all currently sampled regions of the GrIS ablation zone including in the south, south-west, mid-west, north-west and east^9,12–14^. Communities are consistently dominated by two commonly reported glacier algal species, *Ancylonema nordenskioldii* and *A. alaskanum*, with *Cylindrocystis brebissonii* often present at lower abundances^1,4,13–16^. DNA evidence has highlighted glacier algal presence in more interior regions of the ice sheet, above the equilibrium line altitude where melt does not routinely occur^15^, and field surveys have confirmed abundant blooms down to the most marginal reaches of the ice sheet^7^ (despite the apparent “brightness” of such regions in satellite imagery, e.g.,^10,17^).

Snow-pack height, light availability and temperature represent first-order controls on the ability of glacier algal blooms to form and proliferate in any given year^6,8^, with significant interannual variability in bloom magnitude related to the intensity of the melt season in Greenland^8,10,12^. Though glacier algae can be active early in the melt season below rotting snow^18^, blooms do not start in earnest until full ablation of the snowpack exposes the bare ice surface below^12^. Glacier algae then grow within the thin melt-water film that coats melting surface ice crystals of the supraglacial weathering crust^3,12,19^, with doubling times estimated at 3.75–5.5 days in Greenland^4,9^. Given the progressive inland retreat of the snowline as the melt season proceeds, strong spatial patterning develops in algal biomass across the ablation zone, with maximal cell densities found within the most marginal regions that experience the longest ablation periods, decreasing toward the equilibrium line^4,6,7^. With the arrival of winter, the fate of accumulated biomass remains unknown, though observations suggest survival of a minimal seed population for the next ablation period^13,18^.

Despite the apparent oligotrophy, glacier algae can thrive in GrIS surface ice because of their lower absolute cellular macro-nutrient requirements^3,7,20,21^, with multiple nutrient-spiking incubation experiments failing to identify any macronutrient limitation at the point of sampling^2,3,22^. Freshly ablating ice thus appears to provide the nutrients required to support high density blooms and consequently potential future inland expansions of blooms as more of the ice sheet is unlocked by climate warming^20,21,23^. Processes that serve to remove glacier algal cells from the community, i.e., cell mortality and/or physical losses, remain poorly constrained^6,13,24^. While the potential for top-down controls is known (e.g., see refs. ^25–27^), rates of viral lysis and/or fungal infection remain unquantified in Greenland, though^28^ report Chytrid infection rates of 0.6–5.7% from Alaska. Given that glacier algae are non-motile^5^, continued colonisation of fresh bare-ice is likely aided by the slow transport of cells through the weathering crust as melt proceeds^3,24^ or potentially by local aeolian transport^15^. Whilst Stibal et al.^9^ reported precipitation events to export cells from the ice surface, other studies show an increase in glacier algal abundance after storm events^12^. Thus, the impacts of precipitation on glacier algal abundance remain to be fully characterised. Under normal melt conditions, i.e., bright clear sky conditions, physical loss of the total microbial community (all bacteria, algae, and fungi) into the supraglacial stream network is estimated at 30% of the total daily biomass, with smaller bacterial cells depleted more rapidly as compared to larger algal size classes^24^. In melt seasons punctuated by intermittent snowfall events, bloom extent and magnitude are reduced as compared to heavier melt years given burial of the supraglacial ice surface^10^, which presumably restricts the ability of glacier algae to photosynthesise and grow.

Assessment of glacier algal blooms at ice-sheet-wide scales requires approaches to complement spatially limited ground sampling. Several regional studies focused on the SW GrIS have estimated algal abundances and/or extent from optical UAV and satellite imagery on the basis of empirical classification-based relationships between field algal cell counts, field spectra and remotely-sensed reflectance^10,12,29^, or have examined multi-spectral indices employing chlorophyll-diagnostic ‘red edge’ reflectance as proxies for bloom extent^30,31^. Despite limited ground-truthing and difficulties in identifying spectral markers unique to local algal populations, these studies have been able to advance our understanding in interannual variability in GrIS bloom extent and magnitude. They show that more widespread, higher-biomass blooms form in high-melt years when the winter snowpack retreats further and earlier^10,30,31^. They also strengthen evidence of the relationships between algal abundance, darkening of the ice surface and melt^10,30,32^. However, these studies remain confined to regional applications in the SW and tend to lack independent ground validation or uncertainty bounds (Suppl. Note 1). To date, no-ice-sheet-wide remotely-sensed estimation of glacier algal abundance has been developed.

From a modelling perspective, regional models of GrIS glacier algal blooms driven by environmental parameters have been developed by Williamson et al.^6^ (‘GA_BLOOM’) and Onuma et al.^13^. Both model growth as a logistical function based on glacier algal abundance or rates of productivity measured in situ, allowing growth to proceed only during hours when requisite conditions are met. In GA_BLOOM, this is calculated by thresholding environmental time series of i) shortwave-down radiation, as a proxy for photosynthetically active radiation; ii) air temperature, as a proxy for liquid water availability; and iii) snowpack height, to confirm exposure of the bare ice habitat and allow growth under thin snow cover^18^. In contrast, Onuma et al.^13^ simply allow growth to proceed when ice surface temperatures exceed 0 ^o^C. The initial biomass in both models, i.e., the glacier algal seed population size, is derived from back extrapolation of abundance measured in situ across respective study regions, and is reset each year to reflect field observations, which do not yet show interannual accumulation of biomass^13^. GA_BLOOM also includes a daily loss term to represent both physical and biological losses of glacier algae from the supraglacial system (see above). In Williamson et al.^6^ this was expressed as a percentage of the daily population size (kept constant at 10%), and served to decrease algal abundance during e.g., periodic snow fall events. In this configuration, GA_BLOOM accurately replicated absolute cell densities and spatiotemporal patterning in glacier algal biomass measured across the K-transect during the 2016 summer melt season, with modelled biomass showing a strong linear relationship (R^2^ = 0.75) with broadband albedo derived from MODIS satellite observations^6^. Onuma et al.^13^ also successfully recreated glacier algal biomass on the Qaanaaq Ice Cap when applying their model over consistently warm seasons, though model performance decreased in seasons with heterogenous conditions, likely reflecting their use of only ice surface temperature to determine productive hours^33^.

Despite the widespread importance of glacier algae for GrIS surface melt and biogeochemical processes, no study has yet explored the potential distribution of glacier algal blooms across the entire GrIS ablation zone over multiple years. Here we use GA_BLOOM^6^ introduced above, running across a gridded Greenland domain (Methods), to explore key uncertainties in glacier algal bloom ecology and to undertake the first Greenland-wide assessment of likely bloom dynamics over the past two decades. We first conduct sensitivity analyses of key phenological and environmental parameters. Then, we present a Quasi Monte Carlo (QMC) ensemble approach based on (i) our sensitivity analyses and (ii) findings from existing literature, to define plausible parameter spaces for glacier algal growth across Greenland. We use this to estimate the distribution and magnitude of glacier algal blooms around the entirety of Greenland throughout the recent past, which leads us to identify knowledge gaps and outstanding critical research priorities in glacier algal bloom ecology.

## Results and Discussion

### Sensitivity analysis to phenological and environmental parameters

**Fig. 1:**
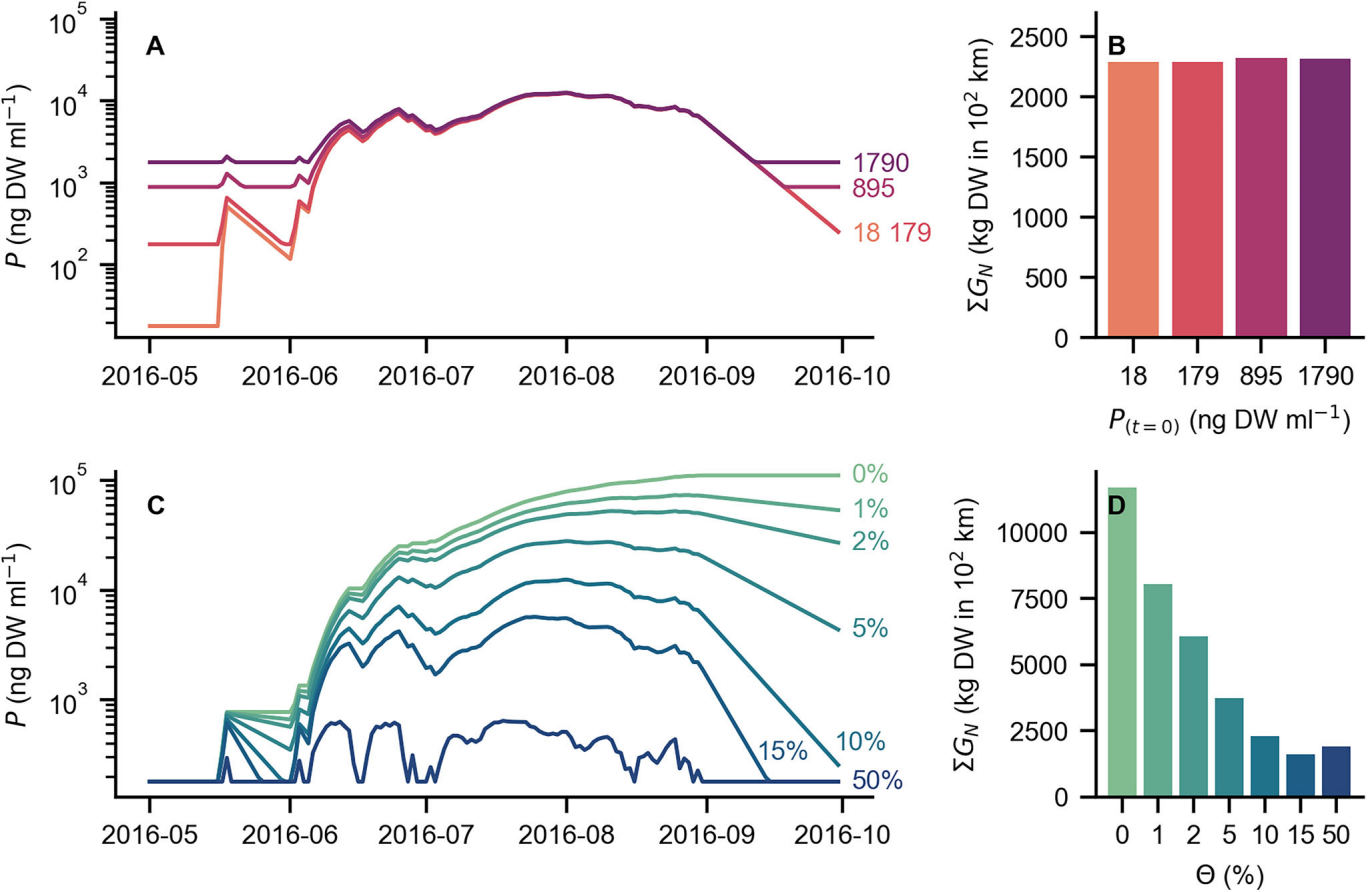
Sensitivity analysis of phenological parameters. **A**, **C** Time series of daily population size in ng DW ml^−1^ at site S6, south-west Greenland. **B**, **D** Total net growth (*ΣG*_*N*_). **A**, **B** Starting populations (*P*_*(t=0)*_) between 18 and 1790 ng DW ml^−1^ (yellow through purple lines). All model runs have a daily population loss term (Θ) of 10%. **C**, **D** Daily population loss as a percentage of existing population (Θ) between 0% and 50% (green through blue lines). All model runs have a *P*_*(t=0)*_ of 179 ng DW ml^−1^.

**Fig. 2:**
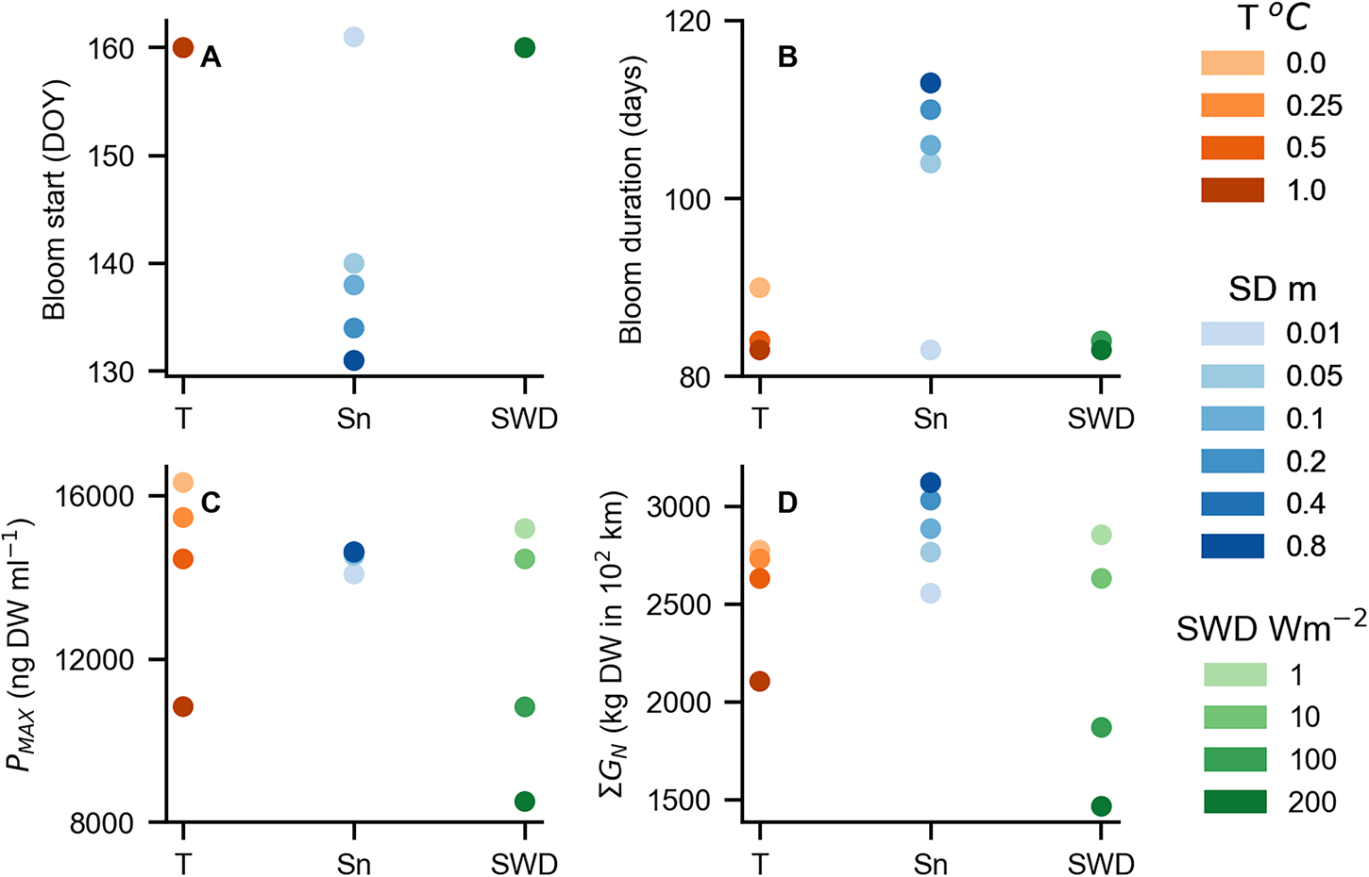
Sensitivity analysis of environmental parameters. **A**–**D** Sensitivity associated with various temperature (*T*), snow depth (*Sn*) and shortwave radiation (*SWD*) thresholds, using the example of site S6 during 2016. All model runs use a starting population (*P*_*(t=0)*_) of 179 ng DW ml^−1^ and population loss term (Θ) of 10%. **A** Variability in the day of year (DOY) of bloom onset. **B** Bloom duration in days. **C** The maximum daily population size achieved during the active bloom season. **D** Total net growth (*ΣG*_*N*_) during the active bloom season, over the 10 km^2^ pixel.

To explore the sensitivity of GA_BLOOM to its constituent phenological (initial seed population size and % daily loss) and environmental parameters (temperature, shortwave radiation and snowpack height), we made multiple simulations for site ‘S6’ in the south-west of the GrIS for summer 2016 to enable comparison with field measurements presented in Williamson et al.^4^ (Methods). Varying the initial seed population size at the start of the ablation season (*P*_*(t=0)*_) by orders of magnitude (18–1798 ng DW ml^−1^) had minimal effects on our two primary metrics, (1) the maximum annual population size achieved (*P*_*MAX*_, Methods; Figs. 1A) and (2) total net glacier algal growth across the season (*ΣG*_*N*_, Methods; Fig. 1B) even during heavy melt years. This likely reflects the rapid increase in algal population sizes permitted by GA_BLOOM at lower glacier algal densities^6^. Our previous use of *P*_*(t=0)*_ = 179 ng DW ml^−1^ was based on back extrapolation of field-population densities measured in southwest Greenland^6^ and reflected the initial presence of active algae from the previous year’s bloom, though at significantly reduced concentrations^13,18^. For comparison, seed population sizes modelled for the Qaanaaq Ice Cap in NW Greenland ranged 3.49–4685 ng DW ml^-1^^13^. Thus, although variability in glacier algal initial population sizes is highly likely around the GrIS and between years, its influence on model behaviour in this context is minimal.

In contrast to *P*_*(t=0)*_ we found a particularly strong regulatory effect of the daily loss term (Θ), expressed as a percentage of total daily population size on glacier algal growth trajectories, with a ~ 2.2 order of magnitude difference in the *P*_*MAX*_ over a range of Θ between 0 and 50 % (Fig. 1C) and an approximately 6-fold difference in *ΣG*_*N*_ (Fig. 1D). When applying the highest Θ (50%), glacier algal biomass modelled for site S6 in 2016 achieved a *P*_*MAX*_ of 0.06 × 10^4 ^ng DW ml^−^^1^, considerably lower than the maximal point abundance of 1.34 × 10^4 ^ng DW ml^−^^1^ recorded at S6 in 2016^4^. Similarly, applying our lower Θ (0%, 1%, 2%) resulted in unrealistic accumulation of glacier algae at site S6 throughout 2016 (ranging 11.11 to 5.25 × 10^4^ ng DW ml^−1^), particularly given the desired spatially averaged nature of GA_BLOOM outputs. Overall, Θ = 10% as used by ref. ^6^ allowed glacier algal communities to grow to realistic abundances observed within our study site, whilst not producing overtly over- or under-estimated spatially averaged densities. Nonetheless, we note that high sensitivity remains: modifying Θ by only a few percent resulted in substantially different population trajectories as shown by the envelope of the 5 and 15 % terms (Fig. 1C), emphasizing the need to quantify physical and biological rates of loss from glacier algal blooms in Greenland.

Considering environmental parameters, varying thresholds of snow depth (*Sn*_*th*_), incoming short-wave solar radiation (*SWD*_*th*_) and hourly near-surface air temperature (*T*_*th*_) that define the duration of the glacier algal daily growth period had distinct impacts on modelled bloom phenology (Fig. 2). The timing of bloom onset and bloom duration were sensitive almost exclusively to *Sn*_*th*_, with a 30-day earlier onset and longer bloom duration when growth was permitted under more snow (0.8 m) as compared to less (e.g., 0.01 m; Fig. 2A, B). In this respect, the temperature and solar radiation conditions required to melt the overlying snowpack at the start of the growth season consistently exceed the minimum temperature and light thresholds needed for algal growth under any scenario tested here, reinforcing the idea that snowpack removal, rather than growth conditions per-se, controls bloom onset in Greenland^4,6,12^. At the end of the melt season, a minor extension of bloom duration (7 days) was apparent when permitting algal growth at lower temperatures, highlighting GA_BLOOM’s sensitivity to late-season cooling when temperatures may decline before snow fall resumes (Fig. 2B).

In contrast to the timing of bloom onset and duration, *P*_*MAX*_ was primarily sensitive to the temperature (*T*_*th*_) and light (*SWD*_*th*_) required to allow growth (Fig. 2C). Raising *T*_*th*_ from 0 to 1 ^o^C resulted in a *P*_*MAX*_ decline from 1.63 to 1.08 × 10^4^ ng DW ml^−1^, while increasing *SWD*_*th*_ from 1 to 200 W m^−^^2^ caused a similar decline from 1.51 to 0.85 × 10^4 ^ng DW ml^−^^1^ for our 2016 test year. Thirty-day differences in the timing of bloom onset and duration from varying *Sn*_*th*_ had minimal effect on *P*_*MAX*_, confirming that for 2016, the length of the daily growth period was largely governed by temperature and solar radiation conditions following snowpack removal. We expect that *Sn*_*th*_ likely remains important for *P*_*MAX*_ for locations/years that are characterised by more heterogenous conditions and/or very late removal of the snowpack. Integrated across the entire growth season, earlier bloom onset and longer durations (reflected here by higher *Sn*_*th*_) resulted in the highest *ΣG*_*N*_, increasing by 23% from our lowest (0.01 m) to highest (0.8 m) *Sn*_*th*_ (Fig. 2D). This reflected previous findings that melt seasons with the longest dark-ice duration and greatest dark-ice intensity are associated with earlier loss of the overlying snowpack^8^. *T*_*th*_ and *SWD*_*th*_ also impacted *ΣG*_*N*_ (Fig. 2D) in a manner analogous to their impacts to *P*_*MAX*_ (Fig. 2C), with the highest values tested here resulting in a 24% and 49% decline in *ΣG*_*N*_, respectively. Total glacier algal growth over the melt season thus showed comparable sensitivity across all three environmental parameters.

### Establishing a Quasi-Monte Carlo ensemble approach

**Fig. 3:**
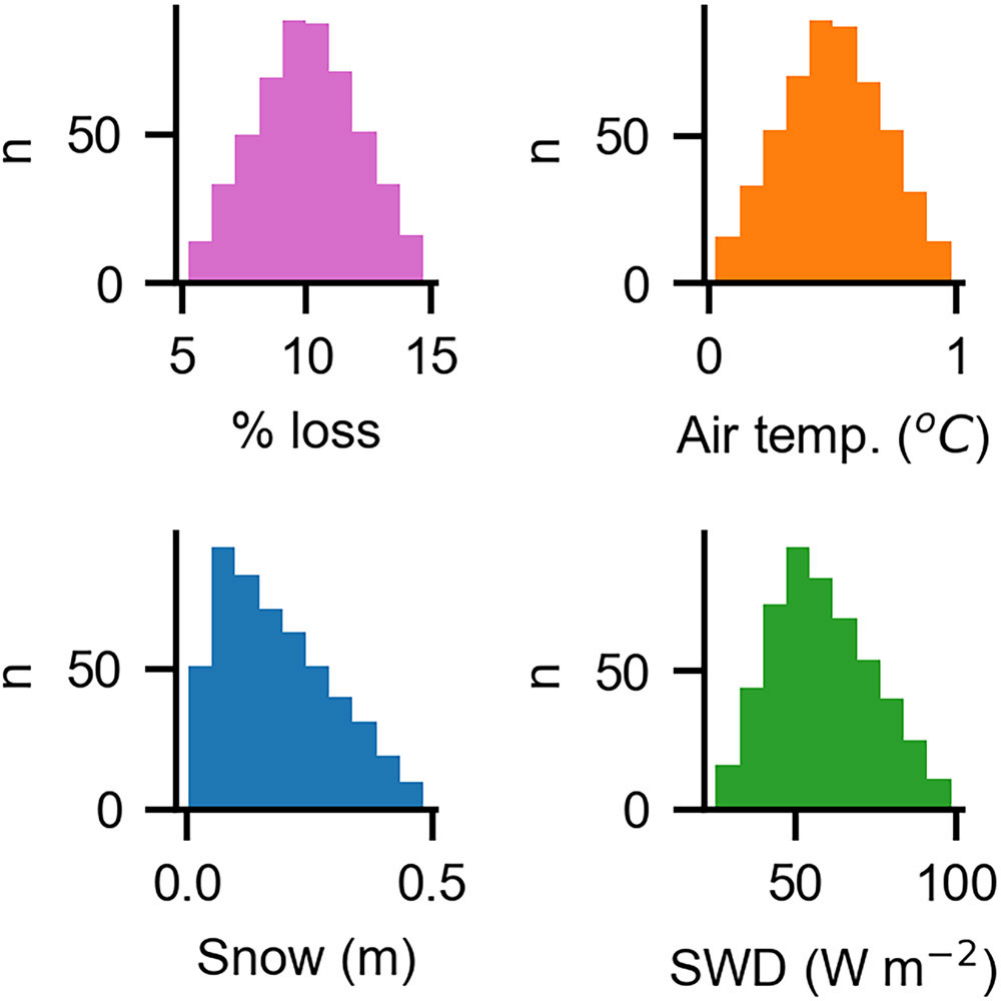
Quasi-Monte Carlo parameter distributions. Histograms of the triangular distribution used to sample each parameter in our Quasi-Monte Carlo ensemble, total *n* = 512.

To explore ice-sheet-wide blooming while acknowledging the wide ranges in plausible model parameters that we identified above, we employed Quasi-Monte Carlo (hereafter QMC) sampling to make multiple simulations over a range of input parameters and then present the spread in those simulations. While regular Monte Carlo sampling is fully random, QMC uses deterministic, quasi-random sequences which effectively sample the entire parameter space with fewer points. In our 512-member QMC ensemble we targeted those parameters shown to significantly influence model outcomes: Θ, *Sn*_*th*_*, T*_*th*_ and *SWD*_*th*_. To do this, we used a conservative approach to define triangular distributions (Fig. 3), allowing to capture across the range of plausible glacier algal bloom trajectories while acknowledging uncertainties in our parameters that remain either poorly or completely unconstrained by field observations. We fixed *P*_*(t=0)*_ at 179 ng DW ml^−1^ across all model pixels in each year, allowing for growth once requisite conditions are met, including in more interior regions of the ice sheet where glacier algal DNA has been found^15^.

**Fig. 4:**
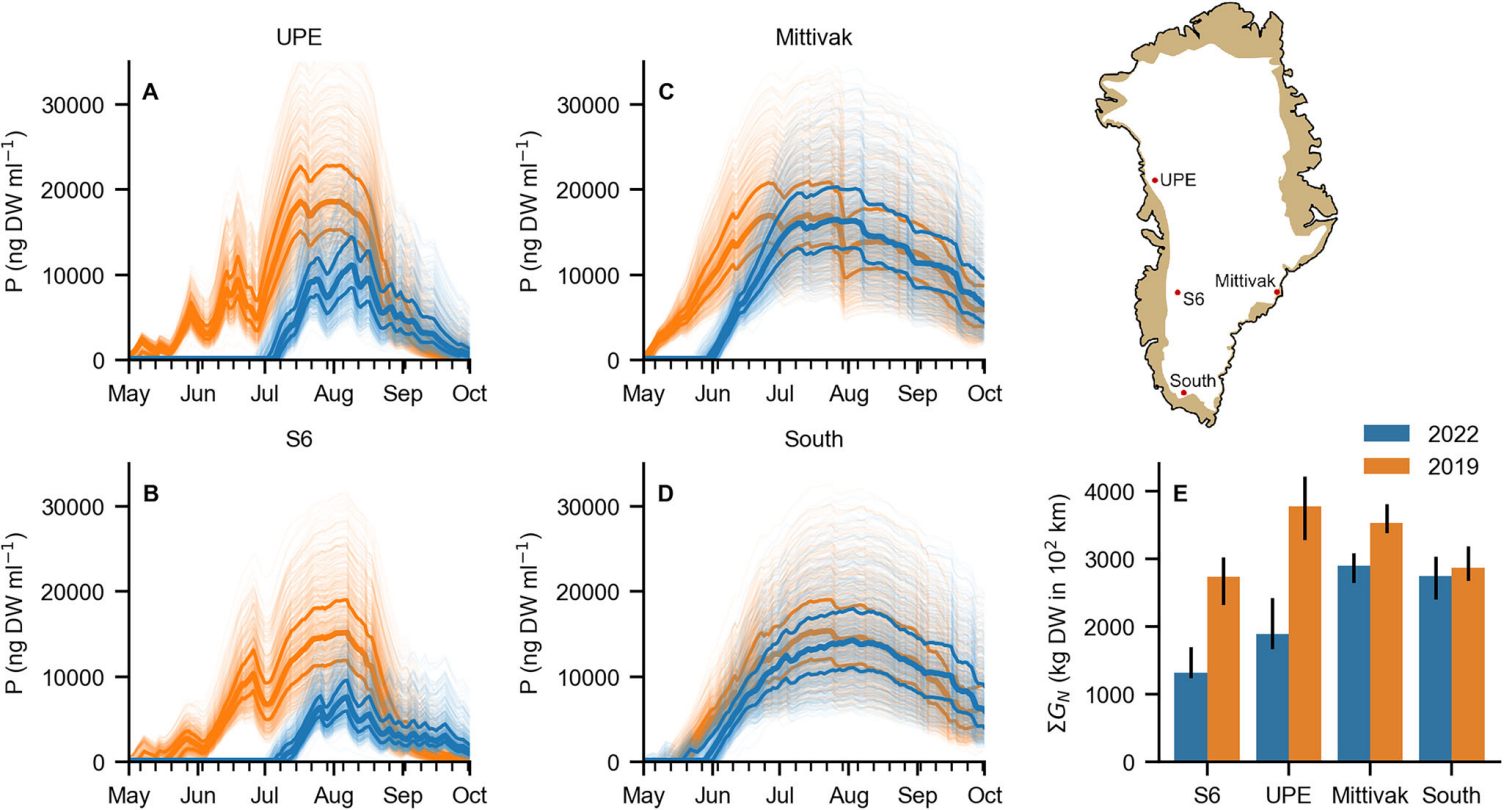
Ensembles of temporal bloom evolution at selected study sites. **A**–**D** Seasonal trajectories of daily bloom size (*P*) at four sites in a recent warmer year (2019, orange) and cooler year (2022, blue). Thin lines show each run in the model ensemble, the thicker lines identify the ensemble median and inter-quartile range. **E** Median total biomass (*ΣG*_*N*_) between the start of algal growth and the last productive day each year, inter-quartile range of the model ensemble shown by black bars. Inset map shows locations of study sites around Greenland.

Representative biomass traces at selected field study sites during a high (2019) and low (2022) melt year (Fig. 4) exemplify the key growth characteristics modelled by our QMC ensemble approach. Plots show the distribution of growth trajectories across our ensemble, highlighting the median and 25^th^/75^th^ quartiles taken forward for calculation of bloom metrics. The timing of bloom onset, *P*_*MAX*_ and *ΣG*_*N*_ can be interpreted here as a function of site latitude, elevation and the relative magnitude of the melt season (high/low). At ice sheet sites S6 (67.1^o^N, 1059 m a.s.l) and UPE (72.8^o^N, 889 m a.s.l), growth initiated ~51–66 days earlier and achieved ~2-times higher *P*_*MAX*_ during our high (2019) as compared to low (2022) melt year, providing a ~ 2-fold greater *ΣG*_*N*_ across the growth season (Fig. 4A, B). At Mittivakkat, a peripheral glacier found at a similar latitude but lower elevation (65.7^o^N, 166 m a.s.l) to S6, growth initiated earlier, particularly in the low melt year, allowing comparable *P*_*MAX*_ to be achieved in both 2019 and 2022, and an overall smaller difference in *ΣG*_*N*_ (Fig. 4C, E). At site South, located at 61.1^o^N and 476 m a.s.l on the ice sheet, highly comparable growth trajectories were achieved in both the low and high melt year (Fig. 4C, E), demonstrating that conditions conducive for glacier algal growth were consistently prevalent at this southerly, lower elevation location. Analyses of our QMC ensemble at site S6 reaffirmed that the most influential parameter shaping model outcomes is Θ: Spearman rank correlation coefficients (ρ) between *ΣG*_*N*_ and input model parameters for our example high melt year (2019) were: *T*_*th*_ = −0.12, *Sn*_*th*_ = 0.31, *SWD*_*th*_ = −0.26, and Θ = −0.89 (Suppl. Figs. 1 and 2).

### Validation against in-situ point measurements

We validated GA_BLOOM against in situ glacier algal abundance datasets that report multiple observations from the same locations and days, allowing to calculate some measure of spread in the observational data (mean ± SD; Methods). This provides a total of 81 unique location/day combinations (integrating a total of 588 ground samples) for us to validate against across the southwestern, northwestern, and southeastern GrIS over multiple years (ranging 2012–2019). However, we note that with a few exceptions^4,9,12^, most datasets have extremely limited replication (e.g., *n* < 5), which is problematic given the highly heterogenous nature of glacier algal colonisation of surface ice at the point scale^5^ in contrast to the 10 km resolution of GA_BLOOM. In addition, several studies do not routinely report if or how their sampling was designed to capture the true spatial average of glacier algae across study regions.

**Fig. 5:**
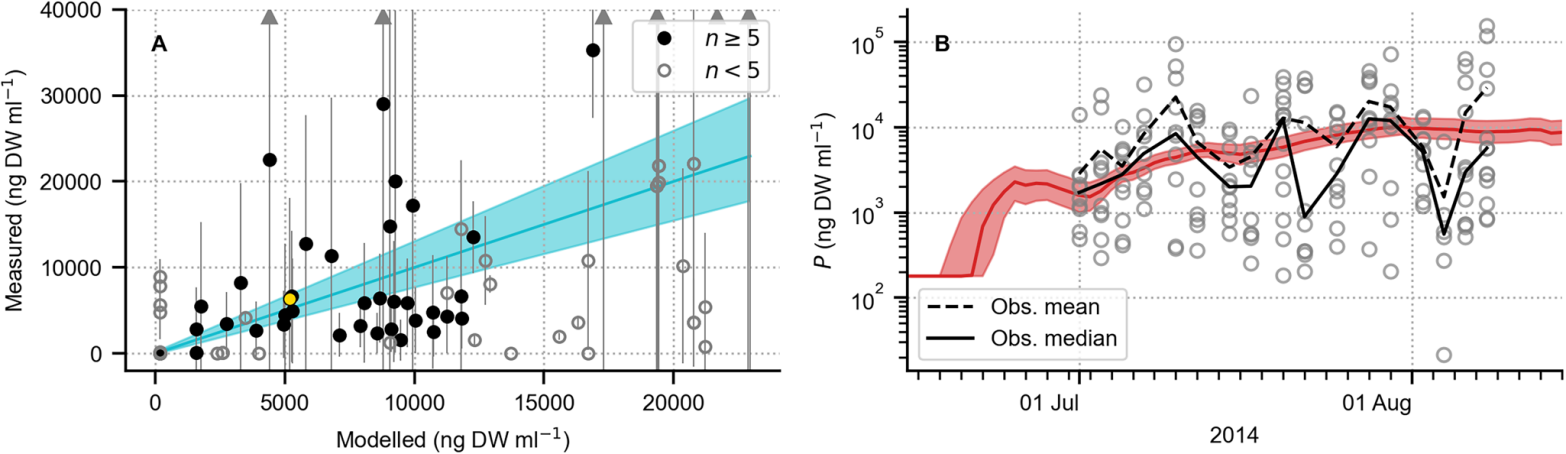
Comparison between modelled and measured biomass concentrations. **A** Modelled biomass (*P*) versus measured biomass, in ng DW ml^−1^ (circles). Closed circles denote observations which are the mean of at least five samples. Hollow circles denote observations with less than five samples. Measured concentrations at site UPE are shown in yellow. Vertical error bars represent the standard deviation of the samples underlying each measurement. Blue bar shows 1:1 line, shading shows the inter-quartile range of the modelled concentrations. Standard deviation ranges which extend beyond the axis limits are denoted by arrows. **B** Modelled biomass (*P*) at site S6, south-west Greenland during the 2014 melt season (red line shows median, shading shows ensemble inter-quartile range) compared to in-situ cell counts made by Stibal et al^9^. converted to ng DW ml^−1^ using a conversion factor of 0.84 (Methods). Grey circles show individual measurements, dashed line shows daily mean, solid line shows daily median.

Model outputs are consistently within the same order of magnitude of mean algal biomass recorded within surface ice across multiple studies, locations and years (Fig. 5A), with strong overlap of modelled inter-quartile ranges and the standard deviations calculated for field datasets, particularly those with higher replication per sampling day (*n* > = 5). Maximal biomass modelled within surface ice ( ~ 2.6 × 10^4^ ng DW ml^−1^) is also lower than maximal point-sample abundances reported across studies in southwestern (1.8 × 10^5^ cells ml^−^^1^^9^), mid-western (5.2 × 10^4^ ng DW ml^−1^, calculated from ref. ^12^), northwestern (4.7 × 10^5^ cells ml^−1^^13^), and eastern (3.6 × 10^5^ cells ml^−1^^14^) Greenland, as expected for the spatially-averaged nature of GA_BLOOM. To our knowledge only^12^ has implemented sampling explicitly designed to capture the spatial average of glacier algal abundance within surface ice (Methods). We note here the strong congruence between the predicted (5185 [3896–7184] ng DW ml^−1^) and mean (6339 ± 9848 ng DW ml^−1^) observed glacier algal abundance for this location and time (Fig. 5A, yellow point), demonstrating GA_BLOOM’s ability to reproduce glacier algal abundance in surface ice beyond the spatial and temporal domains on which it was developed^6^. These comparisons emphasise the need for dedicated ground truth sampling in Greenland that integrates across the significant spatial heterogeneity in glacier algal loading within surface ice, producing rigorous and transparent datasets for model and remote-sensing product validation and calibration.

We also examine the temporal evolution of GA_BLOOM simulations against field observations of Stibal et al.^9^ over the 2014 melt season at Site S6 of the K-transect. In their ground-based study, Stibal et al.^9^ collected *n* = 10 surface ice samples every 2–3 days across a 20 × 20 m area between 1^st^ July and 11^th^ August 2014. Their samples underlying each daily average observation used above (Fig. 5B) illustrate the pronounced small-scale heterogeneity in glacier algal abundance within their sampling area. Considering their daily median and mean values, GA_BLOOM reproduces the magnitude and seasonal evolution of glacier algal loading within surface ice for this location in 2014. Modelled abundance matches initial field counts at the start of observations, with both datasets showing increases in biomass through early July followed by a mid-July moderate decline. During the subsequent period to early August, the high short-term variability in ground observations is not reproduced by GA_BLOOM, which instead captures the broader bloom trajectory through time. Whilst this may indicate a low sensitivity of GA_BLOOM to short-lived, rapid fluctuations in glacier algal loading within surface ice driven by e.g. heavy precipitation events (Introduction), it shows that GA_BLOOM nonetheless recreates the overall magnitude and seasonal trajectory of blooms at given locations. This is evidenced by the strong congruence in *P*_*MAX*_ and *ΣG*_*N*_ calculated here for the mean (*P*_*MAX*_ = 29.05 × 10^3^ and *ΣG*_*N*_ = 3.97 × 10^5^, ng DW ml^−1^) and median (*P*_*MAX*_ = 12.52 × 10^3^ and *ΣG*_*N*_ = 1.98 × 10^5^ ng DW ml^−1^) field observations of Stibal et al^9^., as compared to GA_BLOOM outputs over the same sampling window (*P*_*MAX*_ = 10.30 [8.16–13.11] x 10^3^ and *ΣG*_*N*_ = 2.47 [1.93–3.19] x 10^5^, ng DW ml^−1^). The validity of GA_BLOOM outputs is further qualitatively supported by comparison to remote-sensing retrievals of glacier algal abundance across four sites in the GrIS dark zone over the 2016 and 2017 melt seasons (Suppl. Fig. 3, Suppl. Table 2 and Suppl. Note 1).

### Regional patterning in glacier algal blooms

**Fig. 6:**
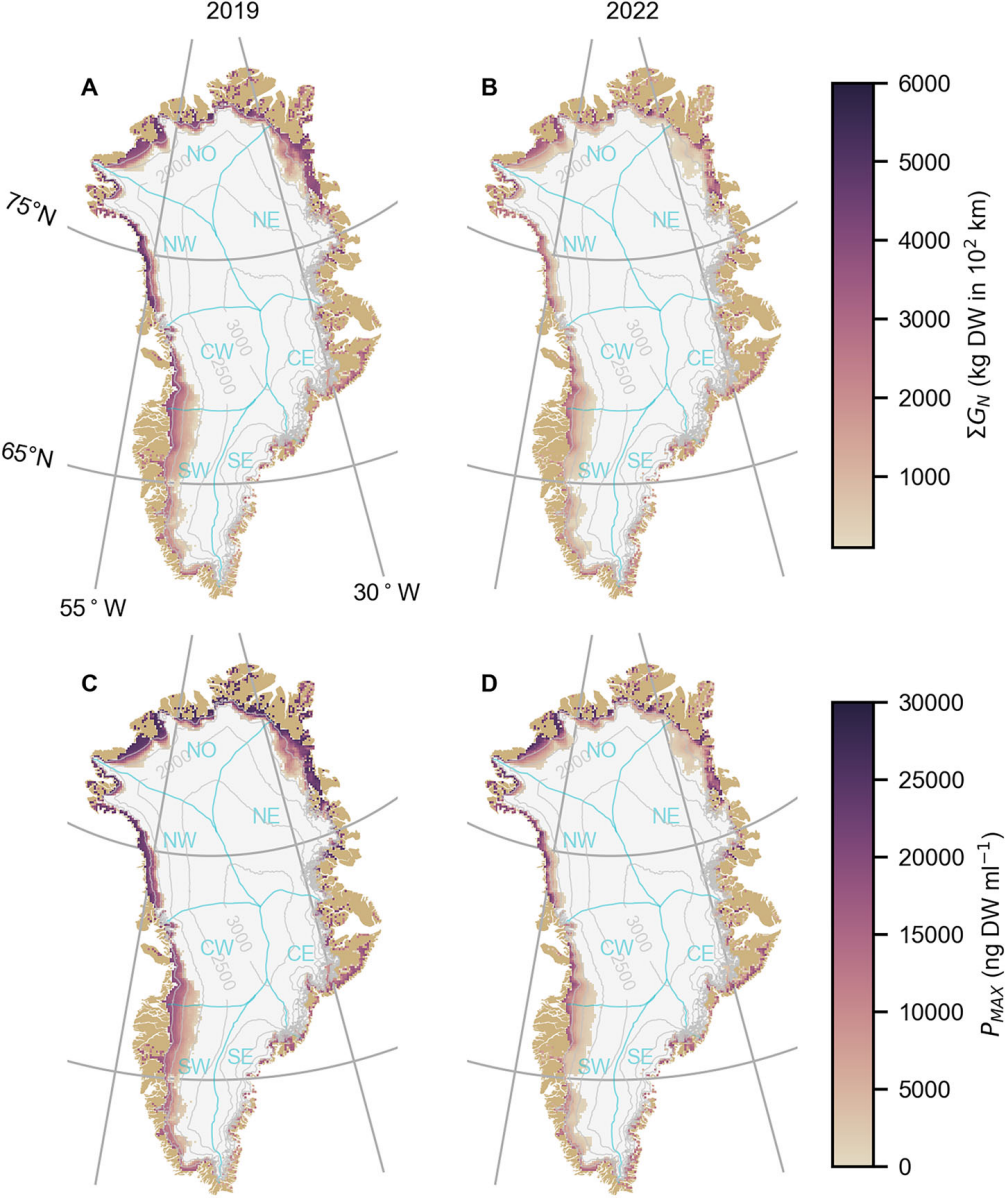
Map of bloom characteristics in cold and warm years. **A**, **B** Total net growth (*ΣG*_*N*_) in 2019 (warm) and 2022 (cold). **C**, **D** As (**A**, **B**), but showing the maximum daily population size *P*_*MAX*_. **A**–**D** All values are calculated from the median of the QMC ensemble in each model cell. Ice sheet regions (cyan) from^49^. Ice sheet surface contours (grey) extracted from ArcticDEM^50^.

**Fig. 7:**
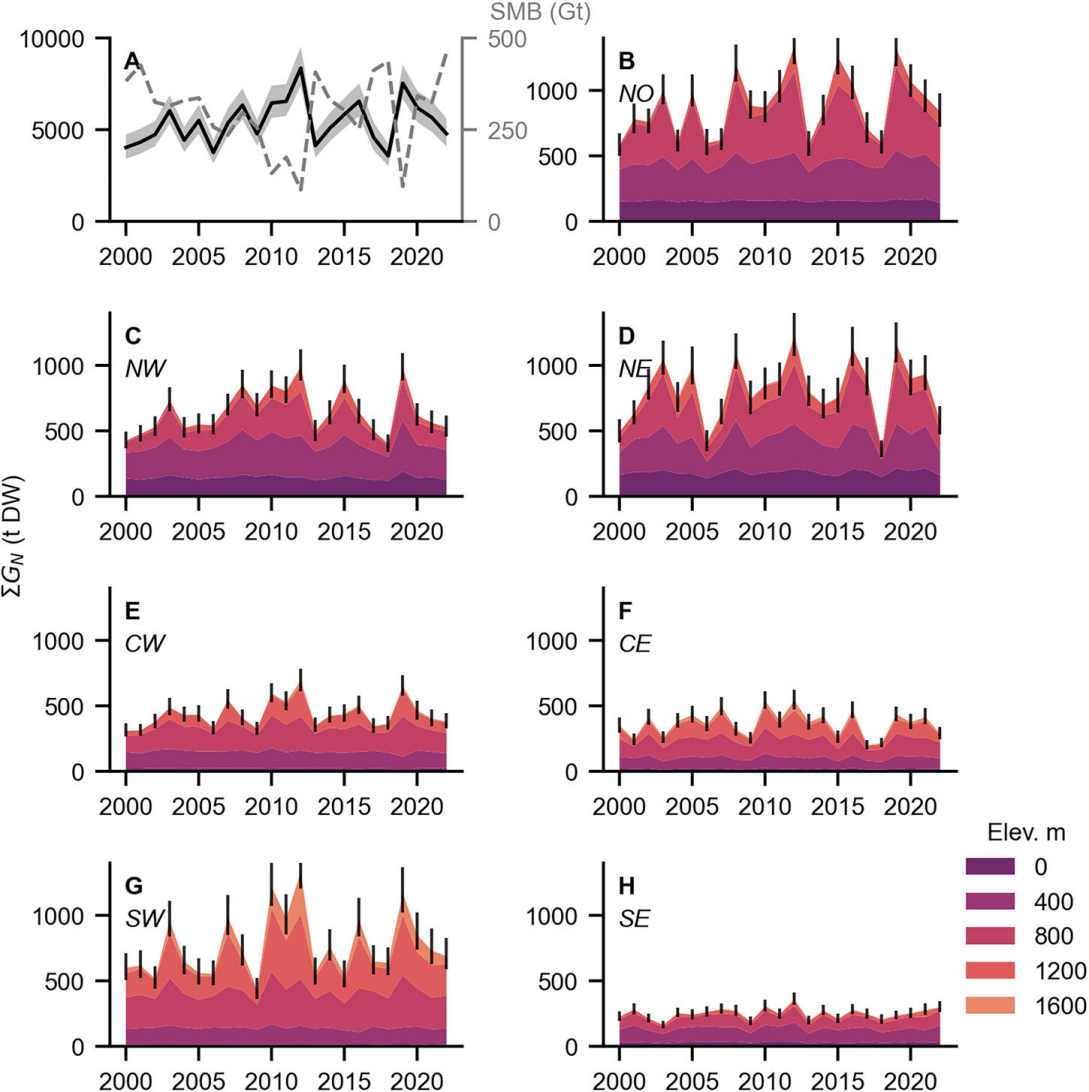
Annual bloom biomass across the Greenland Ice Sheet between 2000 and 2022. **A** Left axis: Total annual net bloom growth (*ΣG*_*N*,_ tonnes dry weight) summed across the Greenland Ice Sheet, peripheral glaciers and ice caps, showing the ensemble median (black line) and inter-quartile range (grey shading). Right axis: Annual surface mass balance over the Greenland ice sheet^51^. **B**–**H** Total annual net bloom growth (*ΣG*_*N*_, tonnes dry weight) by ice sheet sector (Fig. 5) in 400 m elevation bins, excluding peripheral glaciers and ice caps. Vertical black lines show the ensemble’s inter-quartile range in the sum of the sector’s *ΣG*_*N*_.

Site-specific characteristics in growth trajectories (Fig. 4) translate into significant spatiotemporal heterogeneity in the modelled magnitude and extent of glacier algal blooms across Greenland, exemplified by comparison of both the most recent high (2019) and low (2022) surface mass balance years (Fig. 6) and the inter-annual variability across the full study period (Fig. 7). Consistent with field observations^4,6^, maximal biomass occurs at more marginal sites given the longer melt season available for blooms to propagate^4,8^, with biomass decreasing with distance inland from the ice sheet margin. During high melt years such as 2019 (Fig. 6A, C), greater *P*_*MAX*_ and *ΣG*_*N*_ are apparent around Greenland, with a concomitant expansion of blooms further into the ice-sheet interior reflecting migration of the snow line to higher elevations^34^. In contrast, low melt years such as 2022 (Fig. 6B, D) are characterised by lower *P*_*MAX*_ and *ΣG*_*N*_; the inland ice sheet area colonised by glacier algae is reduced, and there is greater spatial heterogeneity in modelled bloom trajectories between our selected time series sites (Fig. 4).

Considering variability by ice sheet sector, modelled glacier algal distribution reflects the extent of the ablation zone and thus the area of melting ice available to support blooms (Figs. 6 and 7). The expansive and relatively flat (between 1 and 2^o^) ablation zone of the south and central west is a known epicentre of glacier algal blooms^1,2,4–7,9,10^. Here, our bloom extents exhibit qualitatively good correspondence with satellite-derived time series of ‘dark ice’ extent, with our largest modelled blooms corresponding to the darkest and most widespread ‘dark ice’ years of 2010–12, 2016 and 2019^8,35^ as well as with the lowest surface mass balance years (Fig. 7A). In contrast, diminutive blooms modelled in southeastern Greenland (SE, Figs. 6 and 7) are consistent with its smaller and steeper ablation zone, with a shorter duration of snow-free conditions, and more limited presence of “dark ice”^35^. Our model shows that while local trajectories of glacier algal growth in southeast Greenland (e.g., on the peripheral Mittivakkat glacier, Fig. 4C) may match (2019) or exceed (2022) those from locations in the southwest (e.g., S6, Fig. 4B), when integrated across ice-sheet sectors, regions with topographically restricted ablation zones such as the SE contribute much less to ice-sheet-wide total glacier algal biomass (Figs. 6 and 7).

Further north, we simulate substantial glacier algal blooms across the northwest sector (NW), where glacier algae are well documented^12,13,36^; the northern (NO) sector, which to our knowledge remains completely unsampled; and the northeastern (NE) sector, where glacier algae have been reported from a single previous study on the A. P. Olsen Ice Cap^9,37^ (Figs. 6 and 7). In the NW and NO sectors there have been +60% and +70% increases in meltwater runoff since 1991, respectively, driven by large-scale Arctic circulation change^38,39^. Underlying this is the 46% expansion in northern Greenland’s (NW + NO) ablation zone, associated with increasingly rapid snowline retreat during early summer^39^. In the NE, the ablation zone includes the narrow, low-elevation ( < 1000 m) ice sheet margin and the middle and lower reaches of the concave Northeast Greenland Ice Stream (NEGIS) catchment, where substantial melt extends far inland up to 1500 m a.s.l.^40^. The NEGIS has increasingly experienced extreme foehn wind events induced by atmospheric river landfalls in NW Greenland, resulting in substantial melt^40^. Our modelling highlights these regions as potential harbingers of extensive glacier algal blooms (Fig. 6) with the modelled capacity to achieve comparable biomass to more southerly regions (Fig. 7). Validation field datasets for northern Greenland thus represent a research priority.

### Greenland-wide bloom dynamics

Greenland-wide, the total modelled glacier algal bloom magnitude varied by a factor of ~3 across our study period, with the lowest total biomass production in 2018 of 3587 tonnes (interquartile range: 3035–4228 t) and the highest in 2012 of 8377 tonnes (7391–9539 t). From this, we estimate the total potential autochthonous organic carbon accumulation by glacier algal blooms across Greenland as ranging 453–1794 tonnes C in 2018 ( ~ 1.51–5.98 kg C km^−^^2^), to 1055–4189 tonnes C in 2012 ( ~ 3.52–13.96 kg C km^−2^); consistent with measured carbon accumulation in Greenland during a glacier algal bloom (up to 14.0 kg C km^2^^24^). While the fate and longevity of this carbon remains unknown, glacier algal blooms support a diversity of secondary producers^2,18,21,25,26^ and a large portion of this organic carbon is likely ultimately routed to en- and sub-glacial systems through the supraglacial stream network^24^. This may represent a significant source of organic carbon for heterotrophs at the ice sheet bed^41^. Although the potential total carbon accumulation associated with Greenland’s glacier algal blooms is modest in the global context^42^, glacier algae nonetheless provide the dominant source of primary production on the ice sheet surface, achieving relatively high rates of productivity^1,4^ despite the ultra-oligotrophic, near-freezing conditions^4,7,21^.

**Fig. 8:**
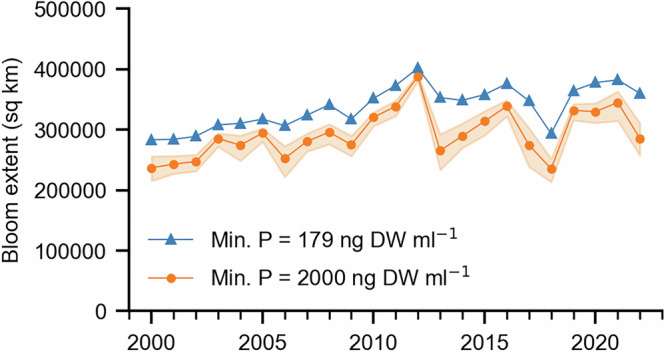
Annual Greenland-wide bloom extent, 2000–2022. All areas of the ice sheet, peripheral glaciers and ice caps are included. Extents shown for two different minimum thresholds of population size *P* at cell which a model cell counts as hosting algal blooms: 179 ng DW ml^−^^1^ (blue triangles) and 2000 ng DW ml^−^^1^ (orange circles). The inter-quartile range in the model ensemble is shown by shading; note that there are no differences in extent across the inter-quartile range at threshold 179 ng DW ml^−^^1^.

Ice-sheet-wide, modelled glacier algal bloom extent was on average 337,800 km^2^ when considering all model cells with *P*_*MAX*_ > *P*_*(t=0)*_, but with more inter-annual variability since 2012 (Fig. 8). Bloom extent increased over our study period by 3724 km^−2^ yr^−1^ (*R*^*2*^ 0.53, *p* < 0.01), although this trend effectively disappears if we only consider model cells with an arbitrarily higher, but still small threshold of *P*_*MAX*_ > 2000 ng DW ml^−1^ (Fig. 8; *R*^*2*^ 0.24, *p* = 0.03). This difference exposes that simulated recent inland expansions of blooms during particularly warm years are transient, with the environmental conditions for growth only met for up to a few days. Nonetheless, in view of projected warming these areas are likely to support bloom growth increasingly often in the coming decades.

## Conclusions

Our findings extend current knowledge on the potential distribution of glacier algal blooms around Greenland previously only derived from field surveys that are significantly restricted in their footprint, or remote-sensing and modelling approaches that remain limited to regional studies to-date. Sensitivity analyses reveal that uncertainties in key phenological and environmental model parameters yield considerable spread in our model ensembles, underscoring the need for improved in situ measurements. Top-down controls and physical removal of glacier algal cells from the ice surface remain particularly poorly constrained yet have a large impact on model outcomes. We also emphasise the current dearth of spatially resolved ground-truth datasets on glacier algal abundance that limits larger-scale model calibration and validation, despite the intensity of their study over the past decade. Nonetheless, we predict a ubiquitous presence of glacier algae around the entirety of Greenland, regardless of the magnitude of the melt season, with longer, larger blooms that expand further into the interior of the ice sheet evident during high-melt years. Modelled bloom magnitude maps to the availability of ablation zone area, with northern sectors of the ice sheet potential harbingers of large-scale blooms that have not yet been sampled. Weak trends of increasing bloom area though time for the lowest glacier algal biomasses tentatively indicate that blooms will expand further into the interior of the ice sheet as/when the long-term equilibrium line altitude rises. This work establishes a series of patterns, mechanisms, and uncertainties for Greenland’s glacier algal blooms to be addressed by future observational and modelling efforts.

## Methods

### Description of glacier algae model

GA_BLOOM^6^ models glacier algal bloom development by calculating potential daily biomass production of the entire glacier algal assemblage as a logistic function of antecedent biomass, constraining this by the proportion of each day meeting environmental thresholds for growth, and updating daily biomass after accounting for proportional losses. The potential daily biomass production of the total glacier algal assemblage (expressed in units of ng dry weight [DW] ml^−^^1^ of meltwater) is estimated based on logistic regression of glacier algal net productivity as a function of biomass, as measured during incubation experiments performed during mid-summer (24 h sunlight and positive temperatures) at site S6 in the south-west of the GrIS^6^. This yields potential daily biomass production *G*_*P*_ as a function of antecedent population size *P* as:

1 $${G}_{P}\left(t\right)=\frac{K}{1+\left(\frac{K-G0}{G0}\right)\exp \left(-r\cdot P\left(t-1\right)\right)}$$

where *K* is the upper asymptote or maximal daily growth potential (2937 ng DW ml^−1^), *G*_*0*_ is the value when *P*(*t*-1) = 0 (796 ng DW ml^−1^), and *r* is the rate constant at which growth potential increases with biomass up to *K* (0.000232). Potential daily production (*G*_*P*_) is then multiplied by the fraction of daily productive hours *H* to calculate the gross daily glacier algal growth, *G*_*G*_:

2 $${G}_{G}\left(t\right)={G}_{P}\left(t\right)\cdot H\left(t\right)$$

H is calculated every day from hourly values of environmental forcing as follows:

$$H(t)\,=\,\frac{\sum (\,(T > {T}_{{th}}) \& ({SWD} > {{SWD}}_{{th}})\& ({SD} < \,{{SD}}_{{th}}))}{24}$$

*T* is the hourly near-surface air temperature (^o^C) which we use as a proxy to describe the presence of liquid meltwater in the habitat; *SWD* is the hourly incoming shortwave solar radiation (W m^-2^) required for photosynthesis; and *SD* (metres) is the snow depth above ice. We explore suitable ranges for each of these environmental parameters (*T*_*th*_*, SWD*_*th*_*, SD*_*th*_) as detailed below.

Net daily growth, *G*_*N*_ is calculated as:

3 $${G}_{N}({{\rm{t}}})={\max} ({0},{G}_{G}({{\rm{t}}})-{{\rm{L}}}({{\rm{t}}}))$$

Where population loss, *L* is expressed as a fraction of the daily population size controlled by the fractional loss term Θ:

4 $${{\rm{L}}}({{\rm{t}}})={{\rm{P}}}({{\rm{t}}})\,\Theta$$

Finally, the daily population size *P* is expressed as:

5 $$P\left(t\right)=P\left(t-1\right)+\,{G}_{G}\left(t\right)-L\left(t\right)$$

Season-long model runs are initialised with a seed population size *P*_*(t=0)*_ which is fixed to a constant value (179 ng DW ml^−1^) for all years of our study based on field observations and our sensitivity analyses (see main text).

To test sensitivities in phenological parameters we fixed environmental thresholds at values similar to Williamson et al.^6^ of *T*_*th*_ = 0.5 ^o^C, *SWD*_*th*_ = 10 W m^−2^, *Sn*_*th*_ = 2 cm, then varied one of *P*_*(t=0)*_ or Θ while leaving the other fixed (at 179 ng DW ml^−1^ or 10% respectively). To test sensitivities in environmental parameters, we ran suites of experiments for each of *T*_*th*_*, SWD*_*th*_ and *Sn*_*th*_ in turn, leaving the other environmental parameters fixed at their mid-range values, *P*_*(t=0)*_ at 179 ng DW ml^-1^ and Θ at 10%. The final choices of phenological and environmental parameters used in the Greenland-wide simulations are discussed in the section ‘Establishing a Quasi-Monte Carlo ensemble approach’ of the Results and Discussion.

### Model forcing

We conducted Greenland-wide simulations with GA_BLOOM between 1 May and 30 September each year over the period 2000 to 2022, using hourly outputs from the regional climate model MAR v. 3.13^43,44^ on a 10 km grid domain forced by ERA-5^45^ at its boundaries. MAR was developed especially for the Polar regions and is coupled to a snowpack energy balance model; it has been extensively evaluated to study Greenland climate and surface mass balance^43,44^. We ran GA_BLOOM in each grid cell of the ice-covered domain (i.e., consisting of the ice sheet, peripheral glaciers and ice caps) independently of other grid cells. We used the MAR variables TT (near-surface temperature), SWD (shortwave-down radiation), SHSN2 (snowpack height above ice) and MSK (ice mask) as inputs to GA_BLOOM.

We note that GA_BLOOM outputs would likely differ if we ran our model using forcing from a different regional climate model. There are appreciable differences in bare ice extent between satellite observations and the two major regional climate models employed over Greenland: MARv3.9 was found to over-estimate satellite-derived bare ice extent by 13% during the period 2001–2017 but to reproduce inter-annual variability quite closely, while RACMO2.3p2 tracked extent more closely but with insufficient inter-annual variability compared to observations^34^. In principle, we may therefore over-estimate bloom magnitude and extent as a function of our environmental forcing by MAR, but we more likely capture real patterns in inter-annual extent. We note that our modelled biomass is conservative with respect to in situ observations (see refs. ^4,5^ Results & Discussion) and our sensitivity analyses exemplify that the dominant source of uncertainty in GA_BLOOM remains unconstrained bloom phenological parameters, not variations in environmental forcing (for which the sensitivity analysis can be considered a partial proxy for employing an alternative forcing scenario).

### Bloom metrics

To summarise glacier algal growth trajectories per grid cell/year we integrated the net daily glacier algal growth (*G*_*N*_) over the duration of the active bloom to derive the total net growth over the melt season (*ΣG*_*N*,_ kg DW per grid cell [10^2^ km]). We calculated active bloom duration as the period between the first and last days on which a given grid cell experienced net algal growth. To allow an intuitive comparison to field measurements, we also calculated the maximum daily glacier algal population achieved each year (*P*_*MAX*_, ng DW ml^−1^). To approximate Greenland-wide total glacier algal biomass production, we summed *ΣG*_*N*_ across the total model domain per year for grid cells demonstrating *G*_*N*_ > *P*_*(t=0)*_. We then estimated the associated carbon (C) accumulation by converting dry weight predictions to units of C across the range of cellular C quotients (106–420 pg C cell^−^^1^) estimated for glacier algae either directly^7,46^ or using allometric or linear biovolume scaling^47,48^. For this we assume a dry weight of 0.84 ng DW cell^−1^ and use a representative 3000 µm^3^ cell^−1^ biovolume.

### Model validation against observations

We primarily validate our model against in-situ (i.e.) field observations of glacier algae. Most in-situ abundance datasets do not have spatial averaging in the sampling design. Often, they deliberately targeted highly colonised ice for sampling or have subjectively sampled across low/medium/highly colonised areas without providing their relative distribution across study sites. Several studies do not provide detailed descriptions of sampling designs. It is thus difficult to translate existing ground datasets into spatial averages to compare with GA_BLOOM outputs. We therefore focus on those datasets which allow us to estimate variability through space and time^4,9,12,13,16,37^ (Suppl. Table 1; Data Availability). These are the only studies which we identified as meeting the requirement of reporting multiple samples from the same location and day so that we could calculate their mean and standard deviation to provide an observation with an associated uncertainty. Observations reported in cells ml^−1^ are converted to units of ng DW per ml^−1^^4^. For each unique location/day observation, we compare to the modelled population size on the day in the pixel of the model domain which intersects the observation’s location. At S6 we also undertook a bloom trajectory comparison with Stibal et al.^9^, for which we present their raw sample values as well as their daily mean and median calculated here.

We undertook a secondary comparison of modelled bloom trajectories against remotely-sensed retrievals of algal biomass at four sites along the SW margin during 2016 and 2017 made by ref. ^31^ (Suppl. Fig. 3 and Suppl. Note 1; Data Availability). The remotely-sensed retrievals are delivered as averages of 7.5 km^2^ areas of interest. No quantification of variance or uncertainty is available in the dataset or the accompanying paper. Our bloom trajectories correspond to the model grid cell which intersects the centre coordinate of each remotely-sensed area of interest (coordinates obtained by personal communication with S. Wang, 13.12.2025).

## Supplementary information

Transparent Peer Review file

Supplementary Material

## Data Availability

Data is available at https://zenodo.org/records/20138073. Field measurements and remotely-sensed retrievals of algal blooms are taken directly from the data repositories lodged alongside their respective study where these exist, otherwise tabulated from the study and/or its supplementary information. The latest MAR model outputs are available at http://ftp.climato.be/fettweis/. Ice sheet mass balance data are available at 10.22008/FK2/OHI23Z. Ice sheet drainage divides are available at 10.7280/D1WT11.

## Code availability

Code for analysis and figure generation is also available at https://zenodo.org/records/20138073.
