# Supplementary material for: Ubiquitous and expanding glacier algal blooms modelled around the Greenland Ice Sheet
Source: Commun Earth Environ. 2026 Jun 16;7(1):613. doi: 10.1038/s43247-026-03758-8 (PMC13400310; doi:10.1038/s43247-026-03758-8)
Supplement: Supplementary file 2 — Supplementary Material [file 43247_2026_3758_MOESM2_ESM.pdf]

Ubiquitous and expanding glacier algal blooms modelled around the Greenland Ice Sheet

Williamson, C. J. and Tedstone, A. J.

Supplementary Information

This file contains:

- Supplementary Figures 1-4
- Supplementary Tables 1-2
- Supplementary Note 1

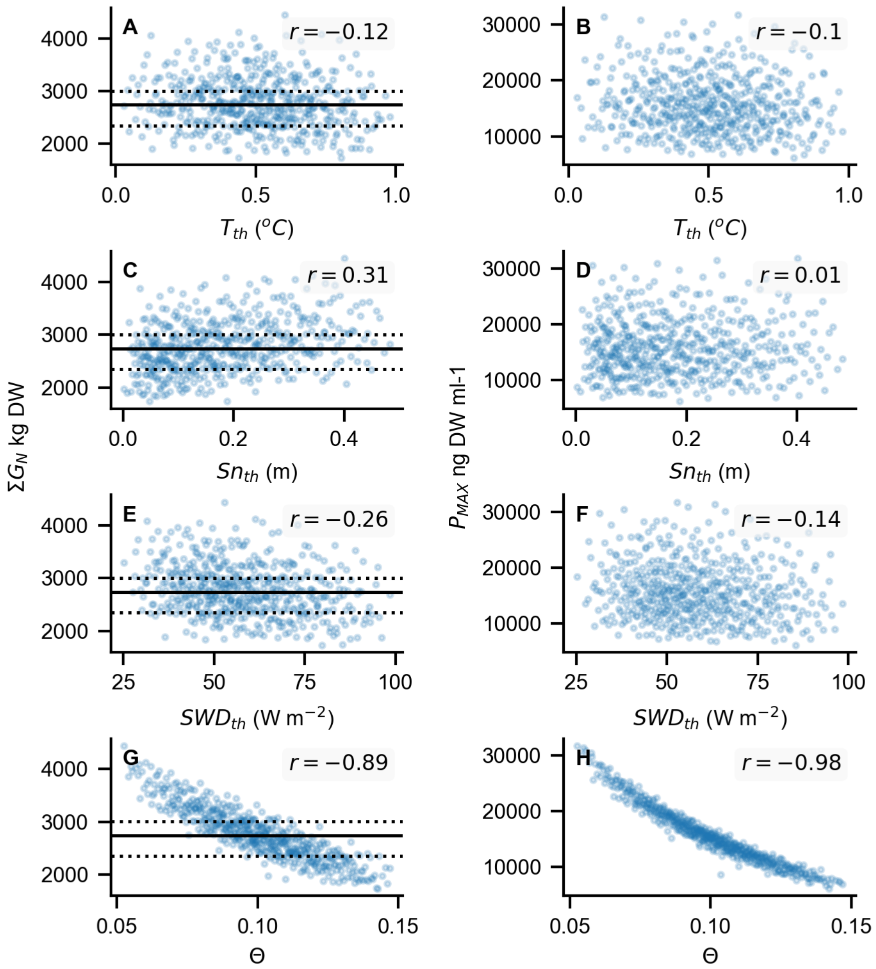

Supplementary Figure 1. Relationships between QMC parameters and modelled blooms at site S6 in a warm year (2019). ΣG_N (left axes) and P_{MAX} (right axes) at S6, plotted against (A,B) minimum temperature threshold T_{th}, (C,D) maximum overlying snow depth threshold Sn_{th}, (E,F) minimum incoming shortwave radiation SWD_{th} and (G,H) fractional population loss term Θ. Each QMC ensemble member corresponds to a single data point. (A,C,E,G) Horizontal black bars correspond to the median (solid lines) and inter quartile range (dotted lines) of ΣG_N across the model ensemble. (A-H) Spearman rank correlation coefficients are indicated in each panel.

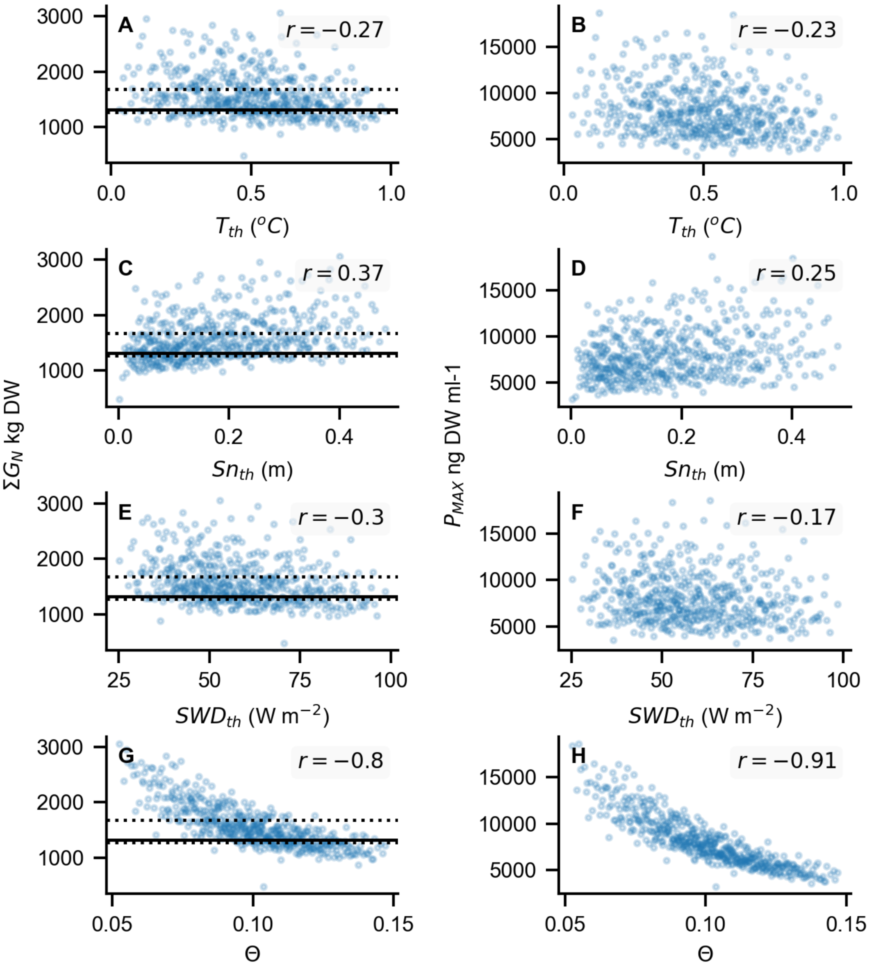

Supplementary Figure 2. Relationships between QMC parameters and modelled blooms at site S6 in a cold year (2022). See caption of Supplementary Fig. 1.

Study	Locality	Obs. year	N. Obs	Samples per obs	S.D.
Included in our validation					
Halbach et al. 2022	S.E. Greenland	2019	2[1]	6 (HE1+2)<br>12 (MIT3-6)	Provided
Onuma et al. 2022	Qanaaq ice cap	2012-14	30	4	Provided
Stibal et al. 2017	S6, SW GrIS	2014	17	9 – 13	Calculated here
Stibal et al. 2017	Various GrIS sites	2013	13	3	Provided
Tedstone et al. 2020	Upernavik, NW GrIS	2018	1	75	Calculated here
Williamson et al. 2018	S6, SW GrIS	2016	10	9 – 13	Calculated here
Williamson et al. 2018	K-Transect	2016	8	4 – 10	Calculated here
Not included in our validation			***Rationale***		
Chevrollier et al. 2022	South	2021	*"The targeted surfaces were chosen to be roughly homogeneous on a wider surface in order to upscale the results for 1 m^2 areas, but are not representative of wider surfaces"*		
Lutz et al. 2014	Mittivakkat	2012	No standard deviation/measure of uncertainty.		
Williamson et al. 2021	P660	2019	Not spatially representative - sampling explicitly targeted ice with a high algal loading.		

[1]Observation at MIT1+2 excluded as original study identifies this as snow, not ice.

Supplementary Table 1. Field studies with datasets of glacier algal abundance in Greenland. Obs. year = the year of observations; N. Obs = the number of unique location/days observations are available for; Samples per obs = the number of replicate surface ice samples reported per unique location/day observation; S.D. = standard deviation, identifying whether this was available within the respective study datasets, or calculated here from these datasets.

Supplementary Note 1

We considered the possibility of assessing our model's performance against remotely-sensed observations which may capture blooming. Limited progress has been made in remote sensing of glacier algal blooms, focussed mainly on southwestern Greenland. These studies can be categorised as either undertaking (i) mapping of glacier algae bloom extent, which concerns most studies in this domain to date, or (ii) direct retrievals of glacier algal bloom abundance from remotely-sensed measurements.

(i) Mapping of bloom extent.

We are aware of three remote sensing studies which have assessed bloom dynamics along the south-western margin of the ice sheet only and by inferring bloom presence without directly retrieving algal abundance.

Using collocated in-situ spectral measurements and algae sampling, Cook et al. (2020) concluded that a uniquely diagnostic spectral biomarker capable of retrieving continuously-varying ice algal abundance is not possible. They were unable to conclusively identify continuously-varying algal abundance from a mixed signal which also incorporated other controls on reflectance such as water content, ice grain size and the presence of other light absorbing impurities. They therefore adopted a classification approach to label bare ice surfaces as either 'clean', 'light algae' or 'heavy algae' according to visual assessment corroborated by microscopy. Yet it is not clear how results from such a classification approach could be systematically compared to our continuously-varying modelled algal abundance at an ice-sheet-wide scale. Furthermore, the maps of surface classification were not archived, so are unavailable for any such comparison.

Wang et al. (2020) aimed to retrieve bloom extent from the MERIS sensor (2004-2011); as MERIS did not have uniquely diagnostic chlorophyll bands they instead investigated several band indices as potential proxy markers. They concluded that *"despite the apparent agreement, further research is required to build a robust relationship between the 2BDA [two-band near-infrared – red] index and algal abundance"*. Furthermore, the post-processed maps of bloom extent are not publicly available. We therefore do not pursue this option as a source of comparison to our model further.

Gerlein-Safdi et al. (2023) proposed that blooms could be mapped using solar-induced fluorescence (SIF) data from the TROPOMI instrument. However, this technique critically relied on Sentinel-3 retrievals of algal bloom extent based on Wang et al. (2018) (introduced below) as training for their SIF algorithm and thus is not an independent approach. Their algorithm showed implausibly large bloom extents appearing and disappearing from one day to another, whether with respect to ground-based knowledge of bloom dynamics (Williamson et al., 2020) or to seasonal remotely-sensed darkening as an indirect assessment of likely bloom dynamics (Feng et al., 2024; Tedstone et al., 2017).

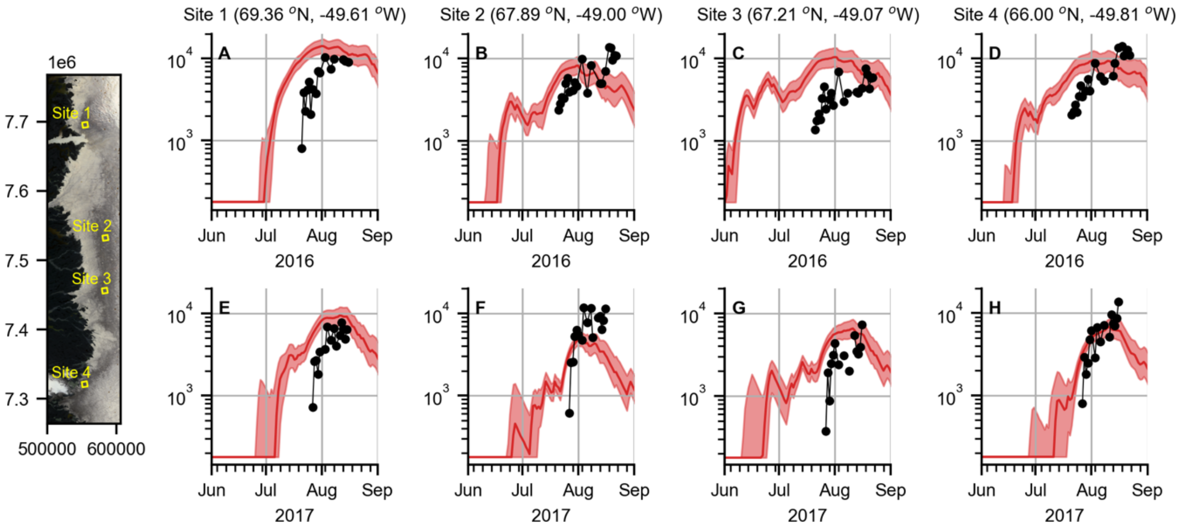

Supplementary Figure 3. Modelled vs remotely-sensed comparison of the temporal development of algal abundance. Time series at four sites along the south-western margin of the Greenland Ice Sheet; sites indicated in location map on left (background: Sentinel-2 composite of cloud-free scenes between 1 July and 30 August 2016). A-H: Remotely-sensed retrievals by Wang et al. (2018) are averages over 7.5 km areas of interest centred over each site's coordinates. GA_BLOOM modelled outputs (red line shows ensemble median, shading shows ensemble inter-quartile range) are from the 10x10 km model cell which intersects the site's centre coordinate.

Year	Site	PMAX Q.50 GA_BLOOM	*PMAX Q.25 GA_BLOOM*	*PMAX Q.75 GA_BLOOM*	PMAX R/S	∑GN GA_BLOOM	∑GN R/S
**2016**	1	14.19	*11.63*	*17.14*	8.67	11.05	7.60
	2	8.24	*6.42*	*10.62*	11.57	4.02	9.16
	3	10.43	*7.98*	*13.55*	6.32	6.04	4.94
	4	9.67	*7.48*	*12.49*	11.99	6.52	9.25
**2017**	1	9.35	*7.33*	*11.88*	6.50	9.07	5.35
	2	4.79	*3.98*	*5.79*	9.80	3.13	9.54
	3	6.53	*5.11*	*8.45*	6.07	5.62	6.07
	4	6.79	*5.43*	*8.47*	11.50	6.14	11.50

Supplementary Table 2. Pmax and ∑GN of modelled (at 25th, 50th and 75th quantiles) vs remotely-sensed (R/S, Wang et al. 2018) glacier algal abundance. Pmax and ∑GN are calculated for biomass traces shown in Supplementary Figure 3 panels A – H over the time interval in which both datasets are available. All data are expressed in units of x10^3 ng DW ml.

(ii) Direct retrievals of glacier algal bloom abundance.

On the Greenland Ice Sheet, only Wang et al. (2018) have undertaken to retrieve algal abundance directly using remote sensing to-date. They used the ratio of Sentinel-3 OLCI bands 11 (708.25 nm) and 9 (673.25 nm) to identify glacier algal abundance. In principle, these bands are uniquely diagnostic of chlorophyll. In practise, they found a Pearson's *r* of only 0.57 between coincident in-situ algal abundance and the field-spectra-derived value of this ratio acquired by Stibal et al. (2017), which suggests that this index may not be uniquely diagnostic of glacier algal abundance. Nonetheless, they developed an exponential relationship between algal abundance and the above OLCI ratio to estimate algal abundance on the basis of Sentinel-3 measurements. We note that no

independent validation of this approach using measurements directly from Sentinel-3 OLCI was undertaken.

Wang et al. (2018) restricted their retrieval of algal abundance to only pixels/areas which were identified as ‘dark ice’ according to a preliminary thresholding approach. We therefore cannot undertake a spatial comparison between their modelled and remotely-sensed bloom extents. However, we can compare their site-specific algal abundance time series at four specific locations within the dark ice area to our model trajectories.

At first order we find overall good agreement between our modelled abundances and Wang et al.’s (2018) remotely-sensed values (Supplementary Fig. 3 and Supplementary Table 2). Earlier in the melt season, satellite retrievals appear to under-estimate algal abundance as compared to both our modelled outputs and field observations in 2016 and 2017 at Site S6, located proximal to Site 3, which showed ~5×10^3 cells ml^{-1} on 16.07.2016, and $2.1 \pm 0.8 \times 10^3$ cells ml^{-1} on 24.06.2017 (Nicholes et al., 2019). These field observations are consistent with our modelled GA_BLOOM outputs but higher than the satellite retrievals if back-extrapolated through time. Later stage bloom abundances are consistent across the two approaches.

We note two specific issues which prevent us from making more quantitative conclusions. First, Wang et al. (2018) provided no indication of either the precision or the uncertainty bounds of their retrievals, which prevents us from understanding whether their substantial day-to-day variability in abundance is real or an artefact of their retrieval approach. Thus, while the remotely-sensed measurements provide a spatially-integrated assessment of bloom magnitude comparable to the scale of our model resolution, we cannot critically interrogate whether our model captures temporal variability in bloom abundance. Second, Wang et al. (2018)’s retrieval of abundances only in pixels which were already identified as ‘dark’ at a given time point means that we have no remotely-sensed evidence of early-season bloom dynamics to compare with our earliest growth phases.

Discussion.

A problem common to all studies described above concerns their ability to retrieve algal abundances along the immediate margins of the south-western region. This gives rise to concern about their ability to capture bloom spatial extents, which might otherwise permit us to qualitatively compare our modelled extents to those mapped by remote sensing. Glacier algae are found at ice-marginal locations in comparable abundances (up to ~9×10^5 cells ml^{-1} proximal to point 660, K-Transect) to further inland sites such as S6 (Williamson et al., 2021), despite satellite products tending to measure brighter ice surfaces nearer the margin (e.g. Cook et al., 2020). While we do not claim to have the answer to this discrepancy, we suspect that these spatial patterns in the remotely-sensed datasets originate in strong differences in surface topographic roughness and, likely, different distributions of algal blooms at the sub-pixel scale. We therefore conclude that these indications of regional-scale disparity between in-situ and remotely-sensed blooms, coupled with a lack of uncertainty bounds on remotely-sensed retrievals, prevent us from confidently using remotely-sensed bloom extents to assess our model’s spatial performance.

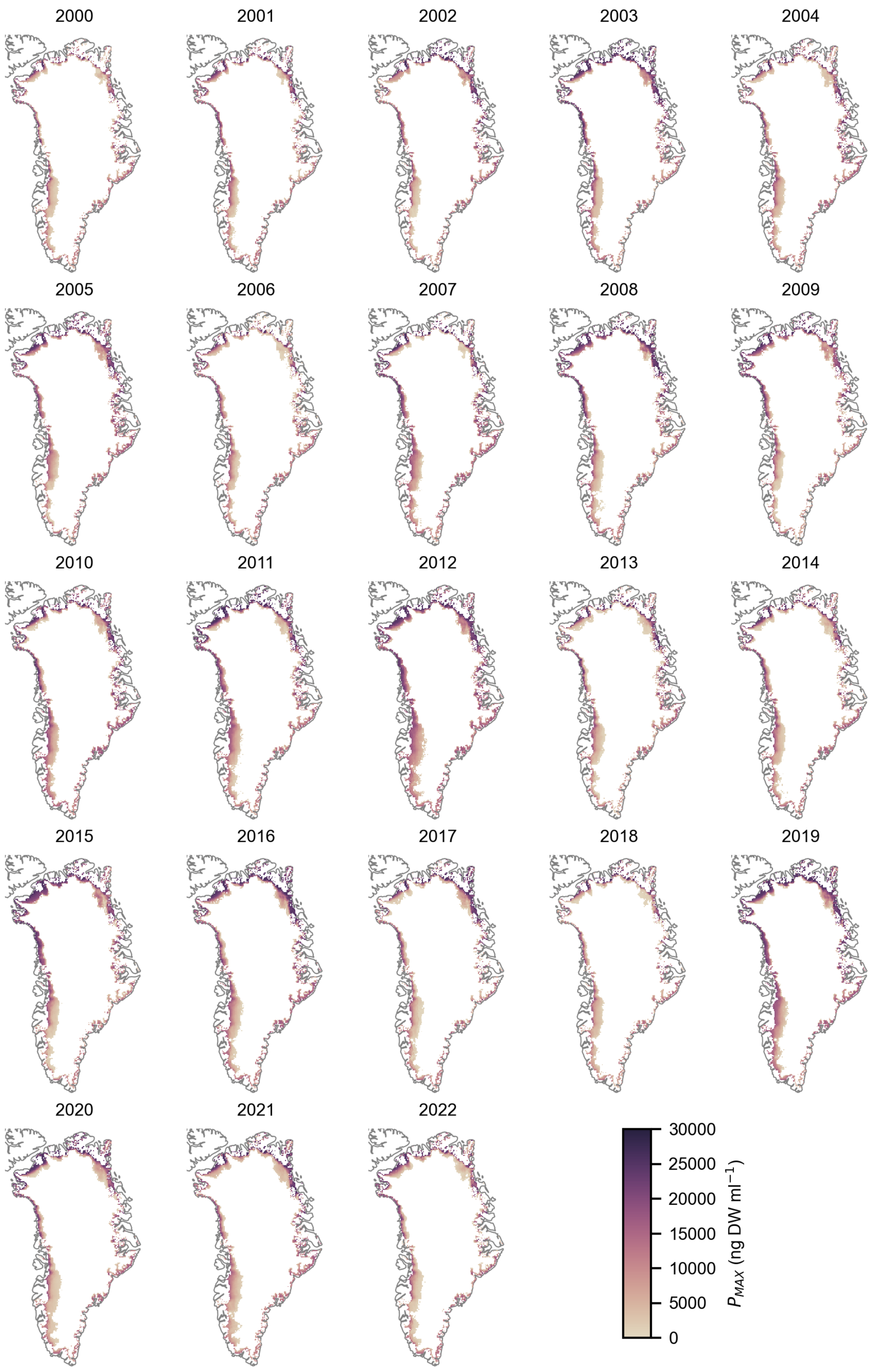

Supplementary Figure 4. Annual ice-sheet-wide maps of maximum population size P_{MAX}.

Supplementary References

1. Cook J*, et al.* Glacier algae accelerate melt rates on the south-western Greenland Ice Sheet. *Cryosphere* **14**, 309–330 (2020).

2. Feng S, Cook JM, Naegeli K, Anesio AM, Benning LG, Tranter M. The Impact of Bare Ice Duration and Geo-Topographical Factors on the Darkening of the Greenland Ice Sheet. *Geophysical Research Letters* **51**, e2023GL104894 (2024).

3. Gerlein-Safdi C, Köhler P, Wang S, Flanner M, Keppel-Aleks G, Frankenberg C. Algae Blooms on the Greenland Ice Sheet Detected Through Solar-Induced Fluorescence. *IEEE Transactions on Geoscience and Remote Sensing* **61**, 1–9 (2023).

4. Halbach L*, et al.* Pigment signatures of algal communities and their implications for glacier surface darkening. *Sci Rep* **12**, 17643 (2022).

5. Lutz S, Anesio A, Villar S, Benning L. Variations of algal communities cause darkening of a Greenland glacier. *FEMS Microbiol Ecol* **89**, 402–414 (2014).

6. Nicholes MJ*, et al.* Bacterial Dynamics in Supraglacial Habitats of the Greenland Ice Sheet. *Front Microbiol* **10**, 1366 (2019).

7. Onuma Y*, et al.* Modeling seasonal growth of phototrophs on bare ice on the Qaanaaq Ice Cap, northwestern Greenland. *J Glaciol* **69**, 487–499 (2023).

8. Stibal M*, et al.* Algae Drive Enhanced Darkening of Bare Ice on the Greenland Ice Sheet. *Geophysical Research Letters* **44**, 11,463–411,471 (2017).

9. Tedstone AJ*, et al.* Dark ice dynamics of the south-west Greenland Ice Sheet. *The Cryosphere* **11**, 2491–2506 (2017).

10. Tedstone AJ*, et al.* Algal growth and weathering crust state drive variability in western Greenland Ice Sheet ice albedo. *The Cryosphere* **14**, 521–538 (2020).

11. Wang S, Tedesco M, Alexander P, Xu M, Fettweis X. Quantifying spatiotemporal variability of glacier algal blooms and the impact on surface albedo in southwestern Greenland. *The Cryosphere* **14**, 2687–2713 (2020).

12. Wang S, Tedesco M, Xu M, Alexander PM. Mapping Ice Algal Blooms in Southwest Greenland From Space. *Geophysical Research Letters* **45**, 11,779–711,788 (2018).

13. Williamson CJ*, et al.* Algal photophysiology drives darkening and melt of the Greenland Ice Sheet. *Proc Natl Acad Sci U S A* **117**, 5694–5705 (2020).

14. Williamson CJ, Turpin-Jelfs T, Nicholes MJ, Yallop ML, Anesio AM, Tranter M. Macro-Nutrient Stoichiometry of Glacier Algae From the Southwestern Margin of the Greenland Ice Sheet. *Front Plant Sci* **12**, 673614 (2021).
