# Supplementary material for: Ubiquitous and expanding glacier algal blooms modelled around the Greenland Ice Sheet
Source: Commun Earth Environ. 2026 Jun 16;7(1):613. doi: 10.1038/s43247-026-03758-8 (PMC13400310; doi:10.1038/s43247-026-03758-8)
Supplement: Supplementary file 1 — Transparent Peer Review file [file 43247_2026_3758_MOESM1_ESM.pdf]

nature portfolio

Peer Review File

Ubiquitous and expanding glacier algal blooms modelled around the Greenland Ice Sheet

Corresponding Author: Dr Christopher Williamson

This file contains all editorial decision letters in order by version, followed by all author rebuttals in order by version.

Version 0:

Decision Letter:

** Please ensure you delete the link to your author home page in this e-mail if you wish to forward it to your coauthors **

Dear Dr Williamson,

Your manuscript titled "Ubiquitous and expanding glacier algal blooms across the Greenland Ice Sheet" has now been seen by 3 reviewers, whose comments are appended below. You will see that they find your work of some potential interest. However, they have raised substantial concerns that must be addressed. In light of these comments, extensive revisions will be required before we can further consider the manuscript for publication. We would, however, be interested in considering a revised version that fully addresses these serious concerns.

We hope you will find the reviewers' comments useful as you decide how to proceed. Should additional work allow you to address these criticisms (that is, either to incorporate the suggestions or provide a compelling argument why the point made by the reviewer is not valid, or relevant to the editorial threshold as outlined below)
AND
meet our editorial thresholds as outlined below, then we would be happy to look at a substantially revised manuscript.
In the following, we list our main editorial concerns.
• Provide a comprehensive description and justification of your modelling approach
• Clearly outline the rationale of the thresholds used in the model
• Provide a robust validation for your model outputs

When resubmitting, please provide a point-by-point response to the reviewers' comments. Please submit your responses as a separate file, distinct from your cover letter where you can add responses to the Editors' comments that you do not want to be made available to the reviewers. Word files are preferred. We recommend that any figures, tables or graphs that are included in the response to reviewers are also included in the main article or Supplementary Information.

Please bear in mind that we will be reluctant to approach the reviewers again in the absence of substantial revisions.

If the revision process takes significantly longer than three months, we will be happy to reconsider your paper at a later date, as long as nothing similar has been accepted for publication at Communications Earth & Environment or published elsewhere in the meantime.

We are committed to providing a fair and constructive peer-review process. Please do not hesitate to contact us if you wish to discuss the revision in more detail.

Please use the following link to submit your revised manuscript, point-by-point response to the reviewers' comments with a list of your changes to the manuscript text (which should be in a separate document to any cover letter), a tracked-changes version of the manuscript (as a PDF file) and any completed checklist:

Link Redacted

** This url links to your confidential home page and associated information about manuscripts you may have submitted or be reviewing for us. If you wish to forward this email to co-authors, please delete the link to your homepage first **

Please do not hesitate to contact us if you have any questions or would like to discuss the required revisions further. Thank you for the opportunity to review your work.

Best regards,

Ilka Peeken, PhD
Editorial Board Member
Communications Earth & Environment
orcid.org/0000-0003-1531-1664

Nicola Colombo, PhD
Associate Editor, Communications Earth & Environment
Consulting Editor, Communications Sustainability

EDITORIAL POLICIES AND FORMAT

If you decide to resubmit your paper, please ensure that your manuscript complies with our editorial policies and complete and upload the checklist below as a Related Manuscript file type with the revised article:

For Manuscripts that fall into the following fields:
• Behavioural and social science
• Ecological, evolutionary & environmental sciences
• Life sciences
An updated and completed version of our Reporting Summary must be uploaded with the revised manuscript
You can download the form here:
https://www.nature.com/documents/nr-reporting-summary.pdf

For your information, you can find some guidance regarding format requirements summarized on the following checklist: (https://www.nature.com/documents/commsj-phys-style-formatting-checklist-article.pdf) and formatting guide (https://www.nature.com/documents/commsj-phys-style-formatting-guide-accept.pdf).

REVIEWER COMMENTS:

Reviewer #1 (Remarks to the Author):

Chris & Andrew use a previously developed logistic growth model for glacier algae to simulate algal bloom across the entire GrIS over the past two decades (2010-2022). Their results show that glacier algal blooms develop annually around the margins of the ice sheet even during low-melt years, albeit with some spatio-temporal heterogeneity, and they show areas with potentially substantial blooms that haven't been directly sampled to date, especially in the N and NW of the ice sheet.

This is a well-written paper that brings interesting and useful data, building on the quite impressive body of the authors' previous research, and fits the scope of Comms Earth & Environment.

I have some questions and comments though I'd like the authors to address before it can be published:

The title of the paper should explicitly say the ubiquitous and expanding algal blooms are modelled/simulated and not observed.

The core of the model (Eq. 1, line 223) is already expressed with concrete numbers which I assume come from the specific incubation experiments mentioned. It would be clearer and more useful if it's shown in an abstract form first and the numbers/values assigned later/below with an explanation maybe? It would certainly help me as a reader not familiar with GA_BLOOM.

I'm not quite sure I understand how the seed population size assumption (292-300) works – surely there must be a functional zero at or below which there is no population growth? I wouldn't expect these in the 'usual' bloom areas, but if the potential growth areas expand inland during high melt seasons, places of functionally zero seed population size might be reached? Or there could be at least some lag before a viable seed population is established -- is it something that the model could simulate?

Also, given that biomass loss comes out as the most significant sensitivity of the model (124) but it's only modelled as a fraction of total biomass, could you discuss the real-world losses in more detail? Is it possible to say whether mechanical or biological losses – which very likely work on different time/space scales -- are the most important? E.g., could the model use precipitation data to simulate biomass loss during heavy rain events (which is something we observed in 2014)?

When you talk about ‘conditions conducive for glacier algal growth’ (e.g. 21), does it also include nutrient availability? E.g., do you assume no difference in nutrient loadings between land- and ocean-terminating sectors of the ice sheet? I understand this is not explicitly modelled but since e.g. P input is linked to the proximity of ice-free land, I would probably expect reduced blooms around the tidewater margins compared to land-terminating. This may be worth mentioning/discussing.

And one last minor thing (182-183): while I agree OC of glacier algal origin could be a substrate for subglacial/proglacial heterotrophs I don’t think it could feed methanogenesis at the bed, since that is constrained to deeper sediment layers where surface OC probably can’t reach, given the relatively short residence time of meltwater in the channelised drainage system; this is also supported by the apparent age and isotopic signature of CH_4 exported from the bed.

I am looking forward to some ground-truthing data from the north though!

Best,
Marek

Reviewer #2 (Remarks to the Author):

This paper present for the 1st time algal blooms modeling over the Greenland ice sheet. It is very original and scientifically relevant. However, as shown by the authors, this first modelling is very dependant of thresholds used for the 3 main variables (temperature, snow depth and solar radiation) and mainly, only model results are shown and discussed in this paper without any robust validation by lack of observations. Fig 7 is far away to be convincing.
Moreover:
- the interannual variability is discussed and suggests that 2012 is the year with the highest algal bloom. In addition to be very dependant of the 3 used thresholds, no physical processes are discussed to explain why it was larger in 2012 vs 2019 for example. Why 2022 vs 2019 is discussed? and not 2018 (lowest) vs 2012 (highest)?
- a daily loss term of 10 % is used to reflect potential mortality. Why 10 %? The results could be also very dependant of this parameter. The mortality could be a function of degrees below 0°C for example? If there is a big snow/cold event in summer, the mortality could be higher than 10 %.

The main drawback of this paper is the lack of validation while it is very original. Satellite based bare ice albedo could be used to detect algal as validation for example. I highly recommend to the authors to find observations to validate the choice of the 3 thresholds as well as the mortality of 10 % and to resubmit a revised version but in the present state, I am sorry to recommend to not accept this paper.

Reviewer #3 (Remarks to the Author):

This study presents a spatiotemporal simulation of glacier algal blooms, which are known to contribute to the darkening of the Greenland Ice Sheet, and investigates the inter-annual trends in algal biomass. A modeling study of glacier algal growth covering the entire Greenland Ice Sheet has not been conducted previously, making this a highly ambitious work. However, several key processes and discussions are missing in the modeling approach, and I consider that a substantial revision of the manuscript is required. I hope the authors will carefully consider my comments below and improve the manuscript accordingly.

Major comments:
1. The manuscript does not mention cryoconite, which is one of the major factors contributing to the darkening of the Greenland Ice Sheet. Previous studies have demonstrated the importance of cryoconite in surface darkening, particularly in the southwestern region (e.g., Wientjes et al., 2011; Chandler et al., 2015; Irvine-Fynn et al., 2021), as well as in the northwestern region (Takeuchi et al., 2014). Furthermore, Hodson et al. (2010) conducted biological modeling of cryoconite on the Greenland Ice Sheet. Because both glacier algae and cryoconite contribute to the darkening processes, the manuscript should include a discussion acknowledging the role of cryoconite.

2. The manuscript should provide clear validation of the temporal variations in biomass simulated by GA_BLOOM. While Williamson et al. (2020) is cited, that paper does not primarily focus on bloom modeling and appears to lack detailed justification for the model equations or validation with observational data, although it’s a great paper. Thus, I strongly recommend that the authors include a time-series comparison between modeled and observed biomass to verify the performance of GA_BLOOM. It is uncommon for studies on algal growth modeling to omit such validation. Furthermore, the growth processes assumed in GA_BLOOM should be described in greater detail. The model uses environmental parameters such as air temperature, solar radiation, snow depth and algal reduction ratio, but the rationale for including these particular factors is not well explained. Are there other environmental variables that may also influence algal growth but are not considered in the model?

3. According to Stibal et al. (2017), extreme precipitation events can significantly wash off glacier algal cells on bare ice surfaces. Because this phenomenon has been observed in the southwestern region of the GrIS, it should be considered as a potential process contributing to the biomass decay term in GA_BLOOM. In connection with Major Comment 2, the manuscript should, at a minimum, include a discussion of precipitation as a possible factor influencing biomass reduction.

4. Satellite observations indicate that the southwestern margin of the Greenland Ice Sheet remains bright, while surface darkening occurs slightly inland (e.g., Wientjes et al., 2011; Wang et al., 2020). In GA_BLOOM, algal growth is assumed to increase under warmer conditions and shallower snow depth. Accordingly, Figure 2 shows higher modeled biomass near the ice margin. This result implies that the current model assumptions may not adequately reproduce the observed spatial distribution of surface darkening on the Greenland Ice Sheet. Nonetheless, this discrepancy offers valuable insight derived from the modeling approach and does not reduce the overall significance of the study. However, the authors should include a discussion addressing this issue.

5. In connection with Major Comments 1–4, I recommend that the simulation domain be restricted to the southwestern part of the GrIS. A regional simulation of glacier algal dynamics is itself novel and of high scientific value; focusing on this region alone would still justify publication. Including other regions, such as eastern or northwestern Greenland, where model validation has not been conducted, introduces substantial uncertainty and may risk presenting scientifically unreliable results. Therefore, I strongly encourage the authors to focus on the southwestern GrIS, perform model validation there, and aim to produce a clear and robust modeling study. A full ice-sheet-scale simulation could then be pursued as a follow-up to this work after publication.

6. In connection with Major Comment 5, if the study focuses on the southwestern part of the Greenland Ice Sheet, satellite-derived estimates of darkened area and algal indices are available (Wang et al., 2020; Feng et al., 2022). A comparison with these previous studies would allow for a more quantitative evaluation of the model's spatiotemporal performance. For instance, it would be valuable to assess whether the simulated biomass trends and spatial distribution correspond to the observed trends and patterns of surface darkening obtained from satellite data.

Specific comments:
L20: Should “2010” be “2000”?

L31: It would be better to include a more detailed description of glacier algae, even if not necessarily in this paragraph. What algal species are involved, and which ones contribute to the surface darkening? Should cryoconite also be mentioned in this study?

L71: Which algal species does GA_BLOOM target? Several glacier algal species, such as Ancylonema nordenskioeldii and Ancylonema alaskana, are known to proliferate on glacier surfaces.

L71: According to the original paper (Williamson et al., 2020), the model focuses on the southwestern region of the Greenland Ice Sheet rather than the entire ice sheet. Therefore, the phrase “the first model of GrIS glacier algal blooms” seems somewhat overstated. As in Onuma et al. you cited, it would be better to clearly specify the region on which the model is based.

L73–77: The technical details described in this section would be more appropriate for the Methods section. Here, the authors should instead provide a concise overview of the biological growth and reduction processes upon which glacier algal models have been developed. As written, it is difficult for readers to understand the fundamental processes underlying existing algal growth models. The authors should review relevant literature and discuss, in particular, the key environmental factors that limit algal proliferation.

L91–92: Prior to evaluating model parameters uncertainty and expanding the simulation to the entire GrIS, it is recommended that the authors validate GA_BLOOM against field observations of algal biomass. It would be important to include this analysis to demonstrate model validity. For instance, time-series biomass data reported by Stibal et al. (2017) from southwestern Greenland could be used for this validation.

Results and Discussion: For readability, I recommend reorganizing this section. Beginning with the Quasi-Monte Carlo ensemble results will likely confuse most readers. The authors should first compare the model outputs with in situ measurements, satellite observations, and meteorological data, clearly articulating the environmental conditions under which the model simulates algal proliferation. Subsequently, the results and discussion of the parameter-sensitivity experiments described in the Methods can be presented.

Quasi-Monte Carlo ensemble approach: This analysis currently obscures the main message and adds redundancy; its necessity should be reconsidered. It seems to function primarily as a rationale for extending simulations to the entire GrIS. Prior to addressing parameter uncertainties at the ice-sheet scale, more fundamental validation and discussion are needed (see Major Comments).

L104–127: This section appears to describe only the model simulation results, with no accompanying discussion or interpretation.

Figure 1: Before presenting this figure, it would be helpful to include a figure similar to Figure 7 that compares modeled and observed biomass. If feasible, a time-series comparison would further strengthen the manuscript. For Figure 1, please clarify what the solid lines in panels (a–d) represent, and describe in the caption what kind of uncertainty the ensemble spread in panels (a–e) corresponds to.

L133–135: Since references 2, 4, and 8 all pertain to the southwestern GrIS, the validity for other regions in Figures 2 and 3

remains uncertain. The model validation appears to rely only on the authors' own studies (Williamson et al. and Tedstone et al.). I encourage the authors to compare their results with those of other independent studies. Furthermore, satellite imagery indicates that the margin of the southwestern Greenland Ice Sheet remains bright, which should be discussed explicitly.

L146–149: To support the validity of the model, it would be beneficial to focus on the southwestern region of the GrIS and include a figure comparing the modeled biomass distribution with satellite observations. If comparison with satellite-derived biomass is not feasible, at least a qualitative comparison with satellite imagery should be considered.

L178–179: Reference 18 pertains specifically to the southwestern GrIS; this should be clearly stated in the manuscript.

L189: Although Pmax is described in the Methods section, a brief explanation here would be helpful for readers' understanding.

L202: "Results and discussion" seems to lack sufficient discussion regarding improvements in field observations.

L206–208: The potential for glacier algal blooms to develop in the inland of the GrIS may also depend on the presence or absence of algal spores in those regions. A brief discussion of the possible origin and dispersal of glacier algae would strengthen the manuscript.

L218: Please clearly state which algal species are represented by the model.

Equation 1: A concise explanation of the constants (2937, 796, –0.000232) should be provided. If these constants were empirically derived from observations in southwestern Greenland, that should be explicitly mentioned. It would also be helpful to define the variable t within this section.

Equation 3: The rationale for selecting these particular environmental parameters to determine the growth flag should be clarified.

Equation 3: It may be more appropriate to use ice surface temperature (or skin temperature) instead of air temperature. If air temperature is intended to serve as a proxy for melt conditions, skin temperature would provide a more physically consistent indicator. Could outputs from the MAR model be used for this purpose?

Sensitivity analysis to phenological and environmental parameters: This section is important for understanding the model's dynamics and limitations. I recommend summarizing the key findings of the sensitivity experiments more concisely and incorporating them into the main text.

L354–358: Please clarify the rationale for determining the range of Θ values used in the analysis.

L358–360: Since the initial biomass of glacier algae at the onset of the bloom season likely varies spatially and interannually, a brief discussion of this point should be added.

L386–398: Because this portion presents validation results and discussion, it would be more appropriate to relocate it to the Results and Discussion section.

Figure 6: Please clarify what the differences in marker color intensity represent.

Figure 7: Axis labels should be added for both axes, and the ranges of the x- and y-axes should be consistent, as they obscure the apparent underestimation of biomass by the model. The blue solid line and shaded area may be unnecessary. The current figure could mislead readers into thinking that the model reproduces the temporal variability of observed biomass.

Figure 7: The conditions of the comparison between observed and modeled biomass should be carefully explained in the text. For example, was GA_BLOOM driven by meteorological data from each observation site? If so, how were those data obtained? Were the model results compared using outputs from the same days as the biomass observations?

L463: Feng "S" et al.

L478: add et al.

Ref:
1. Chandler, D. M., Alcock, J. D., Wadham, J. L., Mackie, S. L., and Telling, J.: Seasonal changes of ice surface characteristics and productivity in the ablation zone of the Greenland Ice Sheet, The Cryosphere, 9, 487–504, https://doi.org/10.5194/tc-9-487-2015, 2015.
2. Feng S, Cook JM, Anesio AM, Benning LG, Tranter M (2023). Long time series (1984–2020) of albedo variations on the Greenland ice sheet from harmonized Landsat and Sentinel 2 imagery. Journal of Glaciology 1–16. https://doi.org/10.1017/jog.2023.11
3. Hodson A, Bøggild C, Hanna E, et al. The cryoconite ecosystem on the Greenland ice sheet. Annals of Glaciology. 2010;51(56):123-129. doi:10.3189/172756411795931985
4. Irvine-Fynn TDL et al. Storage and export of microbial biomass across the western Greenland Ice Sheet. Nat Commun

2021;12:3960.
5. Stibal M et al. Algae drive enhanced darkening of bare ice on the Greenland Ice Sheet. Geophys Res Lett 2017;44(2):11463–71.
6. Takeuchi, N., Nagatsuka, N., Uetake, J., and Shimada, R.: Spatial variations in impurities (cryoconite) on glaciers in northwest Greenland, B. Glaciol. Res., 32, 85–94, https://doi.org/10.5331/bgr.32.85, 2014.
7. Wang S et al. Quantifying spatiotemporal variability of glacier algal blooms and the impact on surface albedo in southwestern Greenland. Cryosphere 2020;14:2687–713.
8. Wientjes, I. G. M., Van de Wal, R. S. W., Reichart, G. J., Sluijs, A., and Oerlemans, J.: Dust from the dark region in the western ablation zone of the Greenland ice sheet, The Cryosphere, 5, 589–601, https://doi.org/10.5194/tc-5-589-2011, 2011.
9. Williamson CJ et al. Algal photophysiology drives darkening and melt of the Greenland Ice Sheet. Proc Natl Acad Sci U S A 2020;117(11):5694–705.

** Visit Nature Portfolio's author and referees' website at www.nature.com/authors for information about policies, services and author benefits**

Communications Earth & Environment is committed to improving transparency in authorship. As part of our efforts in this direction, we are now requesting that all authors identified as ‘corresponding author’ create and link their Open Researcher and Contributor Identifier (ORCID) with their account on the Manuscript Tracking System prior to acceptance. ORCID helps the scientific community achieve unambiguous attribution of all scholarly contributions. You can create and link your ORCID from the home page of the Manuscript Tracking System by clicking on ‘Modify my Springer Nature account’ and following the instructions in the link below. Please also inform all co-authors that they can add their ORCIDs to their accounts and that they must do so prior to acceptance.
https://www.springernature.com/gp/researchers/orcid/orcid-for-nature-research

For more information please visit http://www.springernature.com/orcid

If you experience problems in linking your ORCID, please contact the Platform Support Helpdesk.

Version 1:

Decision Letter:

** Please ensure you delete the link to your author home page in this e-mail if you wish to forward it to your coauthors **

Dear Dr Williamson,

Your manuscript titled "Ubiquitous and expanding glacier algal blooms modelled around the Greenland Ice Sheet" has now been seen by 3 reviewers, and we include their comments at the end of this message. They find your work of interest, but some important points are raised. We are interested in the possibility of publishing your study in Communications Earth & Environment, but would like to consider your responses to these concerns and assess a revised manuscript before we make a final decision on publication.

In particular, while reviewers 1 and 2 provided positive evaluations, reviewer 3 continues to raise substantive concerns regarding the validity of your methodology and the robustness of your conclusions. Therefore, we ask you to either compellingly demonstrate the validity of your model for the whole Greenland Ice Sheet, or soften and caveat your interpretations and conclusions to account for the inherent limitations described by reviewer 3.

We therefore invite you to revise and resubmit your manuscript, along with a point-by-point response that takes into account the points raised. Please highlight all changes in the manuscript text file.

Please submit your point-by-point responses as a separate file, distinct from your cover letter where you can add responses to the Editors’ comments that you do not want to be made available to the reviewers. Word files are preferred. We recommend that any figures, tables or graphs that are included in the response to reviewers are also included in the main article or Supplementary Information.

We are committed to providing a fair and constructive peer-review process. Please don't hesitate to contact us if you wish to discuss the revision in more detail.

Please use the following link to submit your revised manuscript, point-by-point response to the referees’ comments (which should be in a separate document to any cover letter), a tracked-changes version of the manuscript (as a PDF file) and the completed checklist:

Link Redacted
** This url links to your confidential home page and associated information about manuscripts you may have submitted or be reviewing for us. If you wish to forward this email to co-authors, please delete the link to your homepage first **

We hope to receive your revised paper within six weeks; please let us know if you aren't able to submit it within this time so that we can discuss how best to proceed. If we don't hear from you, and the revision process takes significantly longer, we may close your file. In this event, we will still be happy to reconsider your paper at a later date, as long as nothing similar has been accepted for publication at Communications Earth & Environment or published elsewhere in the meantime.

Please do not hesitate to contact us if you have any questions or would like to discuss these revisions further. We look forward to seeing the revised manuscript and thank you for the opportunity to review your work.

Best regards,

Nicola Colombo, PhD
Associate Editor, Communications Earth & Environment
Consulting Editor, Communications Sustainability

EDITORIAL POLICIES AND FORMATTING

We ask that you ensure your manuscript complies with our editorial policies. Please ensure that the following formatting requirements are met, and any checklist relevant to your research is completed and uploaded as a Related Manuscript file type with the revised article.

For Manuscripts that fall into the following fields:
• Behavioural and social science
• Ecological, evolutionary & environmental sciences
• Life sciences
An updated and completed version of our Reporting Summary must be uploaded with the revised manuscript
You can download the form here:
https://www.nature.com/documents/nr-reporting-summary.pdf

Furthermore, please align your manuscript with our format requirements, which are summarized on the following checklist:
Communications Earth & Environment formatting checklist

and also in our style and formatting guide Communications Earth & Environment formatting guide .

*** DATA: Communications Earth & Environment endorses the principles of the Enabling FAIR data project (http://www.copdess.org/enabling-fair-data-project/). We ask authors to make the data that support their conclusions available in permanent, publically accessible data repositories. (Please contact the editor if you are unable to make your data available).

All Communications Earth & Environment manuscripts must include a section titled "Data Availability" at the end of the Methods section or main text (if no Methods). More information on this policy, is available at http://www.nature.com/authors/policies/data/data-availability-statements-data-citations.pdf.

In particular, the Data availability statement should include:
- Unique identifiers (such as DOIs and hyperlinks for datasets in public repositories)
- Accession codes where appropriate
- If applicable, a statement regarding data available with restrictions
- If a dataset has a Digital Object Identifier (DOI) as its unique identifier, we strongly encourage including this in the Reference list and citing the dataset in the Data Availability Statement.

DATA SOURCES: All new data associated with the paper should be placed in a persistent repository where they can be freely and enduringly accessed. We recommend submitting the data to discipline-specific, community-recognized repositories, where possible and a list of recommended repositories is provided at http://www.nature.com/sdata/policies/repositories.

If a community resource is unavailable, data can be submitted to generalist repositories such as figshare or Dryad Digital Repository. Please provide a unique identifier for the data (for example a DOI or a permanent URL) in the data availability statement, if possible. If the repository does not provide identifiers, we encourage authors to supply the search terms that will return the data. For data that have been obtained from publically available sources, please provide a URL and the specific data product name in the data availability statement. Data with a DOI should be further cited in the methods reference section.

Please refer to our data policies at http://www.nature.com/authors/policies/availability.html.

REVIEWER COMMENTS:

Reviewer #1 (Remarks to the Author):

The revised has addressed most of my criticisms and the restructuring/adding the sensitivity analysis/validation section makes a lot of sense.

The introduction has become too long though (almost 4 pages) -- I understand the authors wanted to address all the reviewers' comments but maybe some of that may have stayed in the rebuttal letter?

The main model equation (Eq. 1, line 377) has remained in the numerical form despite the authors promising an abstract form in the response (based on my previous comment) -- could this be fixed?

There is a typo at line 99 (should be "no ice-sheet-wide").

Reviewer #2 (Remarks to the Author):

The responses to reviewers remarks is very convincing and the improvements (structure, validation, discussion/figure about the parameter sensitivities, ...) in the revised version are fine for me.

Reviewer #3 (Remarks to the Author):

Ubiquitous and expanding glacier algal blooms modelled around the Greenland Ice Sheet

Thank you for revising the manuscript. I appreciate the authors' efforts in making substantial changes to address the review comments, and the quality of the paper has improved. However, I found the authors' responses to my comments to be insufficient. Although the authors provided explanations for why certain points were not addressed, I did not find them very convincing. Also, perhaps because the manuscript is now easier to read, I noticed some issues that I had not recognized before. I feel reluctant to raise many additional points again given the considerable effort the authors have already put into revising the manuscript, but please understand that there are still many issues that need to be addressed. I hope the authors will further improve the manuscript by referring to the points below.

Major comments:
1. In my comments on the previous version of the manuscript, I suggested comparing the time series of observed and modeled biomass at several sites across the Greenland Ice Sheet. It is disappointing that in the revised manuscript this comparison is conducted only for the southwestern part of the Greenland Ice Sheet. The authors cite the small number of samples and the lack of spatial representativeness of the observed biomass as reasons for not performing the comparison elsewhere. If so, then doesn't that mean the validity of applying GA_BLOOM to the entire Greenland Ice Sheet cannot be demonstrated? The authors present numerical values comparing observed and modeled biomass outside the southwest, but without a time-series comparison, the validity of the growth model cannot be assessed for the whole Greenland Ice Sheet. In the Appendix, the authors compare satellite-observed and modeled biomass in a time-series manner, but this is also limited to the southwest. While I respect the authors' efforts, shouldn't this paper ultimately restrict the simulation domain to the southwestern region? My conclusion here is also related to what I wrote in Major Comment 2.

2. Looking at Eq. (1), if P(0) is set to 1 ng DW ml^-1, then Gp(0) becomes approximately 796 ng DW ml^-1, implying a 796-fold increase within one hour. Can such a growth rate realistically occur? According to the description in L57, the doubling time is 3.75–5.5 days, which is far from this estimate. With this equation, the growth rate decreases once algal biomass reaches a certain level, so the model may appear to behave realistically in that regime. However, doesn't the formulation have a structural problem in that it allows algae to increase extremely rapidly where there are almost no algal cells present? This behavior can also be seen in Fig. 1. This may be because the model is an empirical equation derived from observations in the dark zone of the southwestern Greenland Ice Sheet, where glacier algae are abundant. Applying this model within the dark zone where glacier algae are already prevalent may be acceptable, but shouldn't we be more cautious about applying it to the entire ice sheet, including inland areas of Greenland where glacier algae are scarce?

3. It would also be better to discuss how the presence of cyanobacteria (cryoconite) and other algal taxa may affect glacier algal biomass. I would like the authors to add a new section and carefully discuss the limitations and outstanding issues of the current model, including the points above. In my previous review, I commented on why the manuscript did not mention cryoconite, which is one of the factors that darken glacier surfaces. The authors responded that they did not address cryoconite because their model does not calculate albedo. However, as the authors also note, glacier algal growth also contributes to surface darkening, so the biological drivers of darkening on the Greenland Ice Sheet likely include both cryoconite and glacier algae. In other words, isn't it common for the two to co-occur on glacier surfaces? Given that real glacier environments contain glacier algae as well as cryoconite and other algae, might differences in community structure in the environment influence glacier algal growth? In addition, in L72 the authors review that parasitism by chytrids can affect

glacier algae, so shouldn't the manuscript also discuss how such organisms may influence glacier algal growth?

Specific comments:
L25: What does "inter-annual variability in melt" refer to—variability in the amount of melt, or in the duration of the melt season?

L27: As the authors themselves recognize, the current model still contains many uncertainties. Therefore, it would be better to avoid presenting such specific numerical values in the abstract.

L35: The model expresses biomass in units of "ng DW mL^-1", but you use "cells mL^-1" here. Could the authors use "cells mL^-1" throughout the paper? Although the conversion is described, "ng DW mL^-1" is not a commonly used unit for comparison with related studies and is not reader-friendly.

L48–49: It seems reasonable—and has been reported in many studies—that when darkly pigmented algae such as Ancylonema bloom, glacier surfaces become darker. However, the claim here appears to differ substantially from previous understanding. Could the authors also add an explanation for why abundant algal cells can be observed even on bright ice? For reference, a recent paper (Traversa et al., 2025) reported a clear correlation between observed Ancylonema biomass and reflectance ratios from satellite and in situ observations. The statement in L48–49 does not seem to describe a universal phenomenon applicable everywhere on the Greenland Ice Sheet.

L75–78: Even if algal biomass increased during periods of heavy precipitation, that does not necessarily prove that precipitation has no effect on flushing algal cells away. For example, it is possible that some cells were transported downstream while new cells were supplied from upstream. Since glacier algae grow on bare ice surfaces, it seems self-evident that intense rainfall or meltwater would make cells more likely to be washed away.

L95–96: This seems to state the opposite of what is written in L48–49. I think readers will be less confused if the manuscript focuses only on more generally applicable facts.

L370: I believe I commented on this before, but which algal species does this model assume for growth? The main glacier algal species include Ancylonema nordenskioldii and Ancylonema alaskanum, and both grow on the Greenland Ice Sheet. Please clarify in the Methods whether Eq. (1) is intended to calculate the biomass of one of these species, or the total biomass of glacier algae.

L156–169: What factors can cause Θ to vary? For example, wouldn't chytrid activity—reviewed by the authors—also affect Θ? Such possibilities should be included in the discussion.

Establishing a Quasi-Monte Carlo ensemble approach: It seems that an explanation of the quasi–Monte Carlo ensemble approach is missing from the Methods section. Also, I think most of L202–211 could be moved to the Methods section.

L208–211: One point to note is that the logic in these lines would apply only if GA_BLOOM is an equation that can be applied across the entire Greenland Ice Sheet. The authors should first demonstrate that the modeled temporal evolution of biomass is applicable to other regions of the Greenland Ice Sheet as well. I am not convinced that Eq. (1), which was empirically derived from observations in southwestern Greenland, can be applied to the Greenland Ice Sheet as a whole, where multiple species co-exist. Shouldn't the algal growth process itself be carefully validated before focusing on tuning model parameters?

L298–301: Looking at Feng et al. (2022), the occurrence frequency of dark ice in the terminal area of southwestern Greenland appears to be relatively low, and the model results (Fig. 7A) do not seem to agree with the satellite observations. In addition, as I mentioned previously, darkening in southwestern Greenland has also been reported to be influenced by cryoconite, so shouldn't this be discussed more carefully?

L325–347: Given that validation across the entire Greenland Ice Sheet is insufficient and the simulations rely on a simple set of assumptions, do the numerical values presented in this subsection really have meaningful significance? I suggest deleting this subsection. At the very least, it would be better to restrict the analysis domain to southwestern Greenland when presenting these numbers.

L354: Improving the glacier algal growth process assumed by the model is also necessary. The manuscript argues—perhaps more than necessary—for improving in situ observational data, but is that truly the main conclusion of this paper? I hesitate to say this, but it felt as though the authors were attributing the inability to validate the model, and the mismatch between the model and observations, to the sampling methods of the in-situ data.

L372: Could you change the unit from "ng DW mL^-1" to "cells mL^-1"? Many related studies express concentration as cell counts (cells mL^-1). Since I have nothing to compare against, I cannot judge how reasonable the biomass values computed by this model are. In particular, I would like to know what the initial value of 178 ng DW mL^-1 corresponds to in cells mL^-1.

L381: Is my understanding correct that H(t) can reach a maximum of 3 if the conditions for each component are satisfied? Please add a clarification.

L406–412: For the entire Greenland simulation, what value did you use for the initial biomass P(0)? What values did you use

for the other parameters, such as the loss rate?

L407: Please specify the names of the MAR physical variables used as inputs to the model. I can guess from Eq. (2), but I'm not certain.

L449–451: I did not understand what this means. Does it mean that you calculated the mean and standard deviation for each observational dataset listed in Table S1 in the Appendix? Since algal biomass varies over time and GA_BLOOM is also a model that simulates the temporal evolution of biomass, is it meaningful to compare using such averaged values? The sampling dates also differ among the observational datasets. Wouldn't it be better to conduct multiple comparisons with time-series observational datasets like Fig. 5b and discuss them carefully, as you did in L260–280?

Figure 5b: The observations from Stibal et al. (2017) are also from southwestern Greenland. Is it not possible to compare the model with time-series biomass observations from other regions?

Supplementary Figure 3: Could you show where Sites 1–4 are located within southwestern Greenland using summer satellite imagery? In my previous comments, I pointed out that the terminus area in southwestern Greenland is white; please show in a figure whether the model is being validated in such regions. This information would also be useful for readers.

Supplementary Figure 3: Since I would like to assess the agreement between the model and observations, could you add the coefficient of determination (R^2) to the figure? Similarly, could you add R^2 to Fig. 5b as well?

** Visit Nature Portfolio's author and referees' website at www.nature.com/authors for information about policies, services and author benefits**

Communications Earth & Environment is committed to improving transparency in authorship. As part of our efforts in this direction, we are now requesting that all authors identified as 'corresponding author' create and link their Open Researcher and Contributor Identifier (ORCID) with their account on the Manuscript Tracking System prior to acceptance. ORCID helps the scientific community achieve unambiguous attribution of all scholarly contributions. You can create and link your ORCID from the home page of the Manuscript Tracking System by clicking on 'Modify my Springer Nature account' and following the instructions in the link below. Please also inform all co-authors that they can add their ORCIDs to their accounts and that they must do so prior to acceptance.
https://www.springernature.com/gp/researchers/orcid/orcid-for-nature-research

For more information please visit http://www.springernature.com/orcid

If you experience problems in linking your ORCID, please contact the Platform Support Helpdesk.

Version 2:

Decision Letter:

** Please ensure you delete the link to your author home page in this e-mail if you wish to forward it to your coauthors **

Dear Dr Williamson,

Your manuscript titled "Ubiquitous and expanding glacier algal blooms modelled around the Greenland Ice Sheet" has now been seen by our reviewers, whose comments appear below. In light of their advice we are delighted to say that we are happy, in principle, to publish a suitably revised version in Communications Earth & Environment.

We request that you fully discuss the limitations of your model framework and account for the uncertainty of your large-scale simulations in the strength of your conclusions.

We therefore invite you to revise your paper one last time to address the remaining concerns of our reviewers. At the same time we ask that you edit your manuscript to comply with our format requirements and to maximise the accessibility and therefore the impact of your work.

EDITORIAL REQUESTS:

Please review our specific editorial comments and requests regarding your manuscript in the attached "Editorial Requests Table".

*****Please take care to match our formatting and policy requirements. We will check revised manuscript and return manuscripts that do not comply. Such requests will lead to delays. *****

Please outline your response to each request in the right hand column. Please upload the completed table with your manuscript files as a Related Manuscript file.

If you have any questions or concerns about any of our requests, please do not hesitate to contact me.

SUBMISSION INFORMATION:

In order to accept your paper, we require the files listed at the end of the Editorial Requests Table; the list of required files is also available at https://www.nature.com/documents/commsj-file-checklist.pdf .

OPEN ACCESS:

Communications Earth & Environment is a fully open access journal. Articles are made freely accessible on publication. For further information about article processing charges, open access funding, and advice and support from Nature Portfolio, please visit https://www.nature.com/commsenv/open-access

At acceptance, you will be provided with instructions for completing the open access licence agreement on behalf of all authors. This grants us the necessary permissions to publish your paper. Additionally, you will be asked to declare that all required third party permissions have been obtained, and to provide billing information in order to pay the article-processing charge (APC).

Please use the following link to submit the above items:
Link Redacted
** This url links to your confidential home page and associated information about manuscripts you may have submitted or be reviewing for us. If you wish to forward this email to co-authors, please delete the link to your homepage first **

We hope to hear from you within two weeks; please let us know if you need more time.

Best regards,

Nicola Colombo, PhD
Associate Editor, Communications Earth & Environment
Consulting Editor, Communications Sustainability

REVIEWERS' COMMENTS:

Reviewer #3 (Remarks to the Author):

Thank you for your response. I regret that the intent of some of my suggestions was not fully understood by the authors. However, since Reviewers 1 and 2 are positive about the revised manuscript, and given the time already invested by both the authors and the editor in the review process, I do not believe that further discussion would be constructive. I therefore have no further comments to add. The manuscript has improved substantially from its original version, and I would like to acknowledge the authors' efforts in revising it.

** Visit Nature Portfolio's author and referees' website at www.nature.com/authors for information about policies, services and author benefits**

Open Access This Peer Review File is licensed under a Creative Commons Attribution 4.0 International License, which permits use, sharing, adaptation, distribution and reproduction in any medium or format, as long as you give appropriate credit to the original author(s) and the source, provide a link to the Creative Commons license, and indicate if changes were made.
In cases where reviewers are anonymous, credit should be given to 'Anonymous Referee' and the source.
The images or other third party material in this Peer Review File are included in the article's Creative Commons license, unless indicated otherwise in a credit line to the material. If material is not included in the article's Creative Commons license and your intended use is not permitted by statutory regulation or exceeds the permitted use, you will need to obtain permission directly from the copyright holder.
To view a copy of this license, visit https://creativecommons.org/licenses/by/4.0/

Response to reviews
Ubiquitous and expanding glacier algal blooms across the Greenland Ice Sheet
Submission 2

Editorial and reviewer comments in black
Author responses in green **with changes in bold**

Your manuscript titled "Ubiquitous and expanding glacier algal blooms across the Greenland Ice Sheet" has now been seen by 3 reviewers, whose comments are appended below. You will see that they find your work of some potential interest. However, they have raised substantial concerns that must be addressed. In light of these comments, extensive revisions will be required before we can further consider the manuscript for publication. We would, however, be interested in considering a revised version that fully addresses these serious concerns.

Many thanks for these reviews. We see that Reviewers 1 and 3 both undertook deep assessments, finding our work 'well-written' and 'ambitious' respectively. In contrast it seems that Reviewer 2 engaged only superficially with our study, evidenced by their minimal and inaccurate comments. Our rebuttals to these comments are provided below.

We hope you will find the reviewers' comments useful as you decide how to proceed. Should additional work allow you to address these criticisms (that is, either to incorporate the suggestions or provide a compelling argument why the point made by the reviewer is not valid, or relevant to the editorial threshold as outlined below)
AND
meet our editorial thresholds as outlined below, then we would be happy to look at a substantially revised manuscript.
In the following, we list our main editorial concerns.
• Provide a comprehensive description and justification of your modelling approach

As we detail in our responses below, our modelling approach was already justified in the original manuscript, but we acknowledge that our previous submission's structure was sometimes difficult to comprehend. We therefore thank reviewer 3 for suggesting **a reorganisation of the manuscript to bring the sensitivity analysis and validation forward into the main text, before the ensemble modelling approach.** We have significantly reorganised our manuscript to this end.

• Clearly outline the rationale of the thresholds used in the model

Much of this editorial concern seems to arise from review 2, which we rebut in detail below. In summary, we emphasise that we do *not* rely on simple fixed thresholds for model parameters. Instead, we define triangular distributions for all parameters shown through sensitivity analyses to have significant impacts on model outcomes, then sample these distributions across 512 individual model runs for each grid cell of our model domain. The distributions themselves are justified by (1) existing knowledge on glacier algal bloom ecology and (2) our sensitivity analyses. **This said, we recognise that our previous**

submission's structure may have obscured some of these details; we hope that our reorganised manuscript addresses these concerns. We now also include a new figure showing our QMC parameter distribution which underlies the thresholds used in each member of the QMC ensemble.

• Provide a robust validation for your model outputs

Our first submission already included validation of our model outputs against available datasets that report more than at least one observation for the same place/day **(see clarified Methods)**. We also note that Reviewer 3 asked us to include validation against the datasets of Stibal et al. (2017). This analysis was already included in the first submission. **In this second submission, we extend our validation against Stibal et al. (2017) further by comparing their temporal bloom dynamics measurements against our modelled timeseries – see Figure 5B and associated text. We have also included a host of other studies from across Greenland in our validation plot, including some additional data collected from around Greenland that we spotted in the depths of Stibal et al. (2017)'s supplementary information during our detailed second review of the literature. We had previously identified the majority of available data but had not included them because of their very low sampling replication (n < 5). For full transparency, we now include these and have added a new Supplementary table 1 outlining each dataset used in validation and the justification for not including others. We have also undertaken a further evaluation of our model outputs against remote-sensing retrievals of glacier algal biomass across four westerly ice sheet sites over the 2016 and 2017 ablation seasons (data from Wang et al. 2018). We include a discussion of the relative merits and complications of such comparisons in Supplementary Note 1.**

Reviewer #1 (Remarks to the Author):

Chris & Andrew use a previously developed logistic growth model for glacier algae to simulate algal bloom across the entire GrIS over the past two decades (2010-2022). Their results show that glacier algal blooms develop annually around the margins of the ice sheet even during low-melt years, albeit with some spatio-temporal heterogeneity, and they show areas with potentially substantial blooms that haven't been directly sampled to date, especially in the N and NW of the ice sheet.

This is a well-written paper that brings interesting and useful data, building on the quite impressive body of the authors' previous research, and fits the scope of Comms Earth & Environment.

Many thanks for this positive review.

I have some questions and comments though I'd like the authors to address before it can be published:

The title of the paper should explicitly say the ubiquitous and expanding algal blooms are modelled/simulated and not observed.

We agree**. The title now reads: Ubiquitous and expanding glacier algal blooms modelled around the Greenland Ice Sheet.**

The core of the model (Eq. 1, line 223) is already expressed with concrete numbers which I assume come from the specific incubation experiments mentioned. It would be clearer and more useful if it's shown in an abstract form first and the numbers/values assigned later/below with an explanation maybe? It would certainly help me as a reader not familiar with GA_BLOOM.

We now include an abstract description of the model in our revised manuscript prior to the formal numerical description to make comprehension easier. We have also revised our description of the initial model used here in our revised introduction.

I'm not quite sure I understand how the seed population size assumption (292-300) works – surely there must be a functional zero at or below which there is no population growth? I wouldn't expect these in the 'usual' bloom areas, but if the potential growth areas expand inland during high melt seasons, places of functionally zero seed population size might be reached? Or there could be at least some lag before a viable seed population is established -- is it something that the model could simulate?

We agree that this is a challenging point. Our rationale for our chosen approach is as follows.

First, we maintain a base seed population for all pixels of the ice sheet as our model requires some biomass present in order to populate growth with the onset of requisite conditions. Seed population sizes are taken from back extrapolation of our field datasets and are consistent with others (e.g. Onuma et al. 2022) from other regions of the ice sheet. We do

not include pixels that only show seed population sizes in any assessment of bloom extent or magnitude – these are effectively zeros for all downstream analyses.

Second, field studies to-date (especially Lutz et al. 2018) which have looked for the presence of glacier algae in regions of the ice sheet not yet experiencing melt (in this instance above the equilibrium line altitude in SW Greenland) have found genetic evidence for their presence. Coupled with widespread evidence from field and/or incubation studies that freshly melting ice provides sufficient nutrients to sustain bloom biomasses (e.g. Williamson et al. 2018, McCutcheon et al. 2021, Millar et al. 2024, Halbach et al. 2025), we conclude that the consensus is that as more of the ice sheet is unlocked by melting, the propagules and nutrients will be available for blooms to initiate. This is matched by the ubiquitous presence of glacier algae in Greenland surface ice wherever melting is occurring according to currently available observations. For these reasons we believe our modelling approach with a base seed population across the Greenland Ice Sheet is justified.

Third, we agree that there may be a lag before populations become established in new inland locations. We suggest that GA_BLOOM already captures this effect by proxy as it finds only minimal, short-term growth in further inland, higher altitude locations during large melt years. In our temporal trend analysis we show that there is only a statistically-significant increase in bloom extent through time when very small glacier algal biomass max. populations are incorporated. This trend disappears if we screen these pixels out. Thus, our temporal analysis with vs without ephemeral blooms already gives some insight into the dynamics of seed populations.

For clarity, we have added more information into our revised introduction on i) DNA evidence for the presence of glacier algae above the ELA in SW Greenland ; ii) sufficient nutrients likely present to sustain blooms in interior ice sheet regions. We have also incorporated discussion on the likely variability in seed population size around Greenland and between years into our section 'Sensitivity analysis to phenological and environmental parameters'.

Also, given that biomass loss comes out as the most significant sensitivity of the model (124) but it's only modelled as a fraction of total biomass, could you discuss the real-world losses in more detail? Is it possible to say whether mechanical or biological losses – which very likely work on different time/space scales -- are the most important? E.g., could the model use precipitation data to simulate biomass loss during heavy rain events (which is something we observed in 2014)?

Yes, we have added more on this into our introduction based on this comment. Our approach is to demonstrate that biomass loss is the most significant term in our model and to perform model simulations accordingly that use as sensible a triangular distribution in potential loss rates (ranging 5-15 %), as reconciled through sensitivity analyses and comparisons of resultant biomass trajectories to known bloom magnitudes from in-situ observations.

We know from e.g. single-cell C/N/P analyses (e.g. Halbach et al. 2025) that glacier algal communities represent a collection of cells all at different stages in their life cycles and physiological states. Viral and/or fungal infection will affect a proportion of the community at

any given time (we are starting to get some numbers on this now, which were included in the original manuscript), with death/lysis the final product of these interactions. We have no evidence yet of temporal dynamism in rates of viral or fungal infections, though we presume there are cycles here that relate to key stages in glacier algal bloom state (initiation, peak, decline). Modelling these would probably be best served with distinct viral/fungal/etc top-down controls, but no field data to drive these are available yet.

In contrast, mechanical losses will relate more to the prevailing melt and/or precipitation regime. The minimal knowledge on these processes is already included in the manuscript (e.g. Irvine-Fynn et al. 2021), **which we now also discuss in the introduction.** These provide some rates of microbial cell export into the supraglacial stream network, highlighting that larger size classes are transported slower and exported at a lower rate than smaller bacterial classes. **This is also included throughout the revised manuscript.**

Re. rain effects, GA_BLOOM does not currently model these, though we do pause glacier algal growth with snowfall, as this results in the most realistic growth trajectories through melt seasons and has also been shown important when modelling snow algal communities (Onuma et al. 2023). Whilst you report in your work (Stibal et al. 2017) that rain resets the ice surface, we have also observed (Tedstone et al. 2020, and during the 2024 melt season at S6) that rain can serve to expand blooms, with high abundances still present after moderate rain events. Rain thus likely has a dose dependent effect, i.e. heavy rain may strip the weathering crust and its cells, whereas moderate rain may aid bloom propagation. We would need more direct measurements to properly include them in our model. At present, we find that pausing growth (but maintaining loss) when snow falls on top of the ice surface produces realistic growth trajectories in the regions we have previously studied. **We now demonstrate how GA_BLOOM integrates over short-term, rapid fluctuations measured in glacier algal loading within surface ice to recreate the magnitude and seasonal trajectory of blooms within locations, by providing a temporal comparison to your point dataset from S6 over the 2014 melt season (Fig 5B).**

When you talk about 'conditions conducive for glacier algal growth' (e.g. 21), does it also include nutrient availability? E.g., do you assume no difference in nutrient loadings between land- and ocean-terminating sectors of the ice sheet? I understand this is not explicitly modelled but since e.g. P input is linked to the proximity of ice-free land, I would probably expect reduced blooms around the tidewater margins compared to land-terminating. This may be worth mentioning/discussing.

As noted above, multiple studies have concluded that there is no strong evidence for inorganic macronutrient limitation of glacier algal communities in situ (or micronutrient [Fe] limitation based on recent presentations at PAM – Jan 2026). In Greenland: Holland et al. (2019) and McCutcheon et al. (2020 - somewhat against the title, but no response to P limitation until after 5 days of closed bottle incubations); in the Alps: Millar et al. 2024; and in Signy Island: Antarctica - Broadwell et al. (in review). Halbach et al (2025) also concluded the same based on single-cell nutrient profiling. Thus, we work on the literature-based assumption that melting surface ice contains sufficient nutrients to support bloom-scale abundances. In this respect, we therefore assume that cells are not macronutrient-limited at the onset of growth. Whilst more P could translate into more growth in different marginal locations as you suggest (and it would be nice to test this), we would need to introduce

Monod or Droop type mechanistic growth trajectories to recreate these impacts within our model. This is certainly the focus of our future work, but may also be tricky for glacial environments and glacier algae given the generally oligotrophic nature of surface ice. **We have enhanced our discussion of this in our revised introduction to draw attention to these dynamics.**

And one last minor thing (182-183): while I agree OC of glacier algal origin could be a substrate for subglacial/proglacial heterotrophs I don't think it could feed methanogenesis at the bed, since that is constrained to deeper sediment layers where surface OC probably can't reach, given the relatively short residence time of meltwater in the channelised drainage system; this is also supported by the apparent age and isotopic signature of CH4 exported from the bed.

Thanks for spotting this. **We have revised to remove reference to methanogenesis.**

I am looking forward to some ground-truthing data from the north though!

Us too!

<u>Reviewer #2 (Remarks to the Author):</u>

This paper present for the 1st time algal blooms modeling over the Greenland ice sheet. It is very original and scientifically relevant.

We thank the reviewer for noting the originality and scientific relevance of our work.

However, as shown by the authors, this first modelling is very dependant of thresholds used for the 3 main variables (temperature, snow depth and solar radiation) and mainly, only model results are shown and discussed in this paper without any robust validation by lack of observations. Fig 7 is far away to be convincing.

There appears to be a major misunderstanding here, we think on the part of the reviewer, though we may have misinterpreted the comment due to problems with its grammar.

We recap that from a methodological perspective, our study has two key novelties: (1) a full sensitivity analysis of all key model parameters, followed (2) by their explicit capture in a quasi Monte Carlo (QMC) ensemble in which we sample parameters across the entirety of the plausible parameter space. It is of course true, as with any model, that the outputs depend strongly on the parameter choices made. Nonetheless, we disagree with the opinion of the reviewer that the results are 'very dependant of thresholds' [sic]: our ensemble inter-quartile range do not span multiple orders of magnitude, and they are within the same order of magnitude with respect to field observations (see next point).

Regarding the comment about 'robust validation', we refer to our responses to reviewer 3 on this subject which explains the further additions we have made in this area. However, we take this opportunity to note that this reviewer has not justified why they are not convinced by our existing analysis nor what validation they would suggest instead, which makes these supposed concerns difficult to address.

Moreover:
- the interannual variability is discussed and suggests that 2012 is the year with the highest algal bloom. In addition to be very dependant of the 3 used thresholds, no physical processes are discussed to explain why it was larger in 2012 vs 2019 for example. Why 2022 vs 2019 is discussed? and not 2018 (lowest) vs 2012 (highest)?

This is incorrect. We provided a dedicated paragraph in the original manuscript linking modelled growth trajectories to major physical controls including the magnitude of the melt season, the latitude and the elevation of sites. We discuss the clear interplay between these three dominant physical controls and how they impact on modelled bloom trajectories across multiple sites (South, S6, UPE, Mittivakkat) around the ice sheet. We opted here to present the most recent high/low melt years rather than the maximum/minimum melt years. We can change the analysis to 2018 and 2012 if desired, but we note that the conclusions will be identical.

- a daily loss term of 10 % is used to reflect potential mortality. Why 10 %? The results could be also very dependant of this parameter. The mortality could be a function of degrees

below 0°C for example? If there is a big snow/cold event in summer, the mortality could be higher than 10 %.

This is incorrect. We do *not* apply a fixed 10% loss term. The loss term varies between 5 and 15% over the 512 model runs which compose our ensemble; its value is sampled from a triangular distribution using our QMC approach.

The main drawback of this paper is the lack of validation while it is very original. Satellite based bare ice albedo could be used to detect algal as validation for example. I highly recommend to the authors to find observations to validate the choice of the 3 thresholds as well as the mortality of 10 % and to resubmit a revised version but in the present state, I am sorry to recommend to not accept this paper.

This comment is baseless. We respond sentence-by-sentence.

> "The main drawback of this paper is the lack of validation while it is very original".
> We are at a loss as to what this sentence means. No further reply possible.
>
> "Satellite based bare ice albedo could be used to detect algal as validation for example."
> It is not possible to detect glacier algae based on albedo alone, because albedo is a broadband measure that provides no means of uniquely detecting biomarkers. See an abundance of literature on this subject. We suggest Shimada et al. (2016), Tedstone et al. (2017) and Cook et al. (2020) as starting points. See also our replies to reviewer 3.
>
> "I highly recommend to the authors to find observations to validate the choice of the 3 thresholds as well as the mortality of 10 % and to resubmit a revised version but in the present state, I am sorry to recommend to not accept this paper."
> As noted above, we perform a full sensitivity analysis on the potential thresholds to be employed here. We do *not* employ one single threshold for any of the parameters the reviewer discusses, instead using triangular distributions for all key parameters in a QMC ensemble approach. We make validation comparisons to all currently available in-situ datasets across authors/years/locations. **See also our new Fig. 3 of the triangular distributions used to sample the parameter space.**

Reviewer #3 (Remarks to the Author):

This study presents a spatiotemporal simulation of glacier algal blooms, which are known to contribute to the darkening of the Greenland Ice Sheet, and investigates the inter-annual trends in algal biomass. A modeling study of glacier algal growth covering the entire Greenland Ice Sheet has not been conducted previously, making this a highly ambitious work. However, several key processes and discussions are missing in the modeling approach, and I consider that a substantial revision of the manuscript is required. I hope the authors will carefully consider my comments below and improve the manuscript accordingly.

We thank the reviewer for taking the time to critique our work, which we believe has led to substantial improvements in its readability.

Major comments:
1. The manuscript does not mention cryoconite, which is one of the major factors contributing to the darkening of the Greenland Ice Sheet. Previous studies have demonstrated the importance of cryoconite in surface darkening, particularly in the southwestern region (e.g., Wientjes et al., 2011; Chandler et al., 2015; Irvine-Fynn et al., 2021), as well as in the northwestern region (Takeuchi et al., 2014). Furthermore, Hodson et al. (2010) conducted biological modeling of cryoconite on the Greenland Ice Sheet. Because both glacier algae and cryoconite contribute to the darkening processes, the manuscript should include a discussion acknowledging the role of cryoconite.

We do not mention cryoconite because this paper is neither about surface darkening nor albedo. This paper's scope is about modelling glacier algal growth in surface ice and so cryoconite is not relevant here. Ultimately, we hope that our model will form a key tool to model glacier algal bloom dynamics and explore impacts on e.g. surface albedo and melt, but our analysis here does not extend to these topics.

2. The manuscript should provide clear validation of the temporal variations in biomass simulated by GA_BLOOM. While Williamson et al. (2020) is cited, that paper does not primarily focus on bloom modeling and appears to lack detailed justification for the model equations or validation with observational data, although it's a great paper. Thus, I strongly recommend that the authors include a time-series comparison between modeled and observed biomass to verify the performance of GA_BLOOM. It is uncommon for studies on algal growth modeling to omit such validation.

GA_BLOOM went through peer review as a major component of Williamson et al. (2020). We recap that we derived model equations from in situ incubation studies on the ice sheet then modelled biomass trajectories through time across the entire 2016 melt season across the K-transect in SW Greenland. We validated these temporal trajectories against in-situ point observations acquired at several points along the K-Transect in the same melt season. Thus we have already established GA_BLOOM's ability to re-create both spatial and temporal growth trajectories of glacier algal biomass on the south-western margin. **We have endeavoured to make this previous work clearer in the Introduction.**

We agree with the suggestion to undertake a time series comparison between modelled and observed biomass, which is now included in the revised manuscript

against the dataset of Stibal et al. (2017) - though we note that this dataset was already used in our previous validation, albeit not in a time series manner. See also our responses to other comments concerning validation below.

Furthermore, the growth processes assumed in GA_BLOOM should be described in greater detail. The model uses environmental parameters such as air temperature, solar radiation, snow depth and algal reduction ratio, but the rationale for including these particular factors is not well explained. Are there other environmental variables that may also influence algal growth but are not considered in the model?

Many environmental parameters impact algal growth, but models are naturally reductive in their approach. At first order, the availability of liquid water (derived from positive air temperatures), sunlight (to drive photosynthesis), and available habitat for growth (i.e. little-to-no overlying snowpack) represent the fundamental pre-requisites for glacier algal growth (e.g. Williamson et al., 2020; Onuma et al., 2022). This matches observations of growth dynamics in surface ice across the cryosphere as well as modelling approaches currently applied to analogous snow algal communities (e.g. Onuma et al. 2018, 2020). **We have updated our Introduction to make the rationale for these parameters clearer.**

3. According to Stibal et al. (2017), extreme precipitation events can significantly wash off glacier algal cells on bare ice surfaces. Because this phenomenon has been observed in the southwestern region of the GrIS, it should be considered as a potential process contributing to the biomass decay term in GA_BLOOM. In connection with Major Comment 2, the manuscript should, at a minimum, include a discussion of precipitation as a possible factor influencing biomass reduction.

We also discuss this particular aspect with Reviewer 1 (Stibal) above. GA_BLOOM does not currently include precipitation effects on glacier algal biomass. We argue that these are poorly constrained. Stibal et al. reported a resetting of the ice surface with precipitation from their 2014 field study, but we have also observed that moderate rain can enhance bloom cover (Tedstone et al., 2020). Thus, more work is needed on this before it can be included in a model. **We have added specific discussion of precipitation dynamics within the introduction of our revised manuscript as well as in our time-series comparison to Stibal et al. (2017) in our now front-loaded validation section.**

4. Satellite observations indicate that the southwestern margin of the Greenland Ice Sheet remains bright, while surface darkening occurs slightly inland (e.g., Wientjes et al., 2011; Wang et al., 2020). In GA_BLOOM, algal growth is assumed to increase under warmer conditions and shallower snow depth. Accordingly, Figure 2 shows higher modeled biomass near the ice margin. This result implies that the current model assumptions may not adequately reproduce the observed spatial distribution of surface darkening on the Greenland Ice Sheet. Nonetheless, this discrepancy offers valuable insight derived from the modeling approach and does not reduce the overall significance of the study. However, the authors should include a discussion addressing this issue.

We first recap that our study is about glacier algal growth, *not* darkening or albedo. The two are linked, but not in a straightforward relationship that we or others have been able to identify.

We nonetheless thank the reviewer for raising this point. They may be interested that our view is rather the inverse: we have demonstrated previously that glacier algae are found at ice-marginal locations in comparable abundances (up to ~9 x 10^5 cells ml^{-1} proximal to point 660, K-Transect) to further inland sites such as S6, despite satellite products indeed tending to measure brighter ice surfaces nearer the margin (Williamson et al. 2021). We therefore rather take the view that it is satellite albedo products that are unreliable for inferring glacier algal growth in such locations. **Please see our new Supplementary Note 1 where we expand on this topic in more detail.**

5. In connection with Major Comments 1–4, I recommend that the simulation domain be restricted to the southwestern part of the GrIS. A regional simulation of glacier algal dynamics is itself novel and of high scientific value; focusing on this region alone would still justify publication. Including other regions, such as eastern or northwestern Greenland, where model validation has not been conducted, introduces substantial uncertainty and may risk presenting scientifically unreliable results. Therefore, I strongly encourage the authors to focus on the southwestern GrIS, perform model validation there, and aim to produce a clear and robust modeling study. A full ice-sheet-scale simulation could then be pursued as a follow-up to this work after publication.

We disagree, for the following reasons.

i) We have already undertaken a regional study in SW Greenland using GA_BLOOM (Williamson et al. 2020, PNAS). This ice-sheet-scale simulation is therefore the follow up to that work, as per the reviewer's suggestion.

ii) Validation here against NW Greenland *is* included (Fig. 7, yellow point with represents the mean across n = 75 ice surface samples) and we have added in data from other regions into the revised manuscript, though we note the very low replication in these studies (n < 5) that is unlikely to capture the true spatial average or glacier algal loading within surface ice, already somewhat evident by the large standard deviations reported for those sites.

iii) All GrIS field sites sampled so far have the same fundamental populations, dominated by the two dominant *Ancylonema* species. Further sampling from across the rest of the cryosphere also tends to support this conclusion. In view of the apparent oligotrophy which suggests that spatially-varying nutrient availability is not a key control on bloom growth, then biologically these blooms are the same at first order, with environmental factors as the key determinant of bloom extent. On this basis we therefore see no reason to restrict the modelling to a certain region only.

iv) It is common to use models to produce testable hypotheses about the distribution/magnitude of natural phenomenon. We argue that it is more useful to make potential biomass products available to the wider community, allowing them to target subsequent field sampling to priority areas. This study may provide justification for future sampling in new areas of Greenland.

v) We do not present our results as definitive conclusions on Greenland's blooms, but instead we use our model to identify key knowledge gaps that need attention and key regions of Greenland that require ground truth sampling.

6. In connection with Major Comment 5, if the study focuses on the southwestern part of the

Greenland Ice Sheet, satellite-derived estimates of darkened area and algal indices are available (Wang et al., 2020; Feng et al., 2022). A comparison with these previous studies would allow for a more quantitative evaluation of the model's spatiotemporal performance. For instance, it would be valuable to assess whether the simulated biomass trends and spatial distribution correspond to the observed trends and patterns of surface darkening obtained from satellite data.

As we note above, albedo measurements do not equal glacier algal abundance. Similarly, there are specific problems with the supposedly unique biospectral markers. Thus, darkness indices do not provide a means of uniquely validating our model's spatial performance. **However, we do now undertake a temporal comparison with the selected sites presented in Wang et al. 2018. See our new Supplementary Note 1.**

Specific comments:
L20: Should "2010" be "2000"?

Many thanks, amended

L31: It would be better to include a more detailed description of glacier algae, even if not necessarily in this paragraph. What algal species are involved, and which ones contribute to the surface darkening? Should cryoconite also be mentioned in this study?

We have added more details on assemblage structure in Greenland. As previously noted, cryoconite is not relevant here, nor is this study focussed on surface darkening.

L71: Which algal species does GA_BLOOM target? Several glacier algal species, such as Ancylonema nordenskioeldii and Ancylonema alaskana, are known to proliferate on glacier surfaces.

It is based on total community abundance. Both these species are present across Greenland **– more information is included in the revised manuscript.**

L71: According to the original paper (Williamson et al., 2020), the model focuses on the southwestern region of the Greenland Ice Sheet rather than the entire ice sheet. Therefore, the phrase "the first model of GrIS glacier algal blooms" seems somewhat overstated. As in Onuma et al. you cited, it would be better to clearly specify the region on which the model is based.

Added as requested and this paragraph has now been redrafted to provide an integrated assessment of both modelling approaches which are highly complementary.

L73–77: The technical details described in this section would be more appropriate for the Methods section. Here, the authors should instead provide a concise overview of the biological growth and reduction processes upon which glacier algal models have been developed. As written, it is difficult for readers to understand the fundamental processes underlying existing algal growth models. The authors should review relevant literature and discuss, in particular, the key environmental factors that limit algal proliferation.

We would draw the reviewers' attention to the original preceding paragraphs that sequentially relay information and associated literature on i) dominant environmental controls on blooms and spatiotemporal patterning in bloom phenology (paragraph 2); ii) bottom-up and top-down controls on blooms (paragraph 3); and iii) knowledge available from regional remote sensing studies (paragraph 3). The paragraph you note here is itself then dedicated to modelling approaches to-date. **We have added additional information into these preceding paragraphs in the revised manuscript to further highlight controls on bloom phenology, and have redrafted the paragraph noted here to synthesise across the modelling approaches of Williamson et al. (2020) and Onuma et al. (2023). We believe that the information presented on modelling approaches is very relevant and important context for readers of this modelling study.**

L91–92: Prior to evaluating model parameters uncertainty and expanding the simulation to the entire GrIS, it is recommended that the authors validate GA_BLOOM against field observations of algal biomass. It would be important to include this analysis to demonstrate model validity. For instance, time-series biomass data reported by Stibal et al. (2017) from southwestern Greenland could be used for this validation.

This validation was already included in the original manuscript, including all Stibal et al. (2017) data, but as noted above, this was not relayed as a time-series. **This is now included in the revised manuscript as well as a detailed supplementary discussion regarding remote-sensed glacier algal abundance across SW Greenland (Wang et al. 2018) and associated comparisons.**

We agree with the reviewer's comment below that our sensitivity analyses and validation should come upfront at the start of the results/discussion. **We have reorganised the manuscript accordingly.**

Results and Discussion: For readability, I recommend reorganizing this section. Beginning with the Quasi-Monte Carlo ensemble results will likely confuse most readers. The authors should first compare the model outputs with in situ measurements, satellite observations, and meteorological data, clearly articulating the environmental conditions under which the model simulates algal proliferation. Subsequently, the results and discussion of the parameter-sensitivity experiments described in the Methods can be presented.

We agree with the reviewers comment and have **reorganised our manuscript**, placing our sensitivity analyses and QMC ensemble approach into the results/discussion so that the rationale and approach are much clearer before the major results are presented.

Quasi-Monte Carlo ensemble approach: This analysis currently obscures the main message and adds redundancy; its necessity should be reconsidered. It seems to function primarily as a rationale for extending simulations to the entire GrIS. Prior to addressing parameter uncertainties at the ice-sheet scale, more fundamental validation and discussion are needed (see Major Comments).

We think that there has been some misunderstanding here. Please see our responses to this reviewer's major comments where we show that the fundamental validation e.g. at the regional scale has already been done.

Incorporating a QMC ensemble approach is critical to assign uncertainty/sensitivity bounds to our model outputs and is a more sophisticated modelling approach regardless of whether we are running this regionally or at the ice sheet scale. The reviewer is correct, we would not want to run the model at the ice sheet scale without an ensemble approach to simulate a range of trajectories in potential bloom outcomes. We are not sure how this approach "obscures the main message and adds redundancy" – this is a very common approach in modelling studies.

L104–127: This section appears to describe only the model simulation results, with no accompanying discussion or interpretation.

We disagree. This paragraph contextualises the model outputs against major physical forcings of site latitude, elevation and the relative magnitude of the melt season, presenting evidence across a series of sites around Greenland to illustrate these points.

Figure 1: Before presenting this figure, it would be helpful to include a figure similar to Figure 7 that compares modeled and observed biomass.

We agree and have reordered our results/discussion (see also earlier responses).

If feasible, a time-series comparison would further strengthen the manuscript.

This has now been added into the revised manuscript – please see earlier comments

For Figure 1, please clarify what the solid lines in panels (a–d) represent, and describe in the caption what kind of uncertainty the ensemble spread in panels (a–e) corresponds to.

Figure 1, now Figure 4 in our resubmission, shows the full results of the QMC ensemble at four different sites. Per the original caption, the thinner lines show each model run from the ensemble, while the thicker lines identify the median and inter-quartile range. All relevant information is already in the caption.

L133–135: Since references 2, 4, and 8 all pertain to the southwestern GrIS, the validity for other regions in Figures 2 and 3 remains uncertain. The model validation appears to rely only on the authors' own studies (Williamson et al. and Tedstone et al.). I encourage the authors to compare their results with those of other independent studies. Furthermore, satellite imagery indicates that the margin of the southwestern Greenland Ice Sheet remains bright, which should be discussed explicitly.

We make the following remarks:

1. Concerning the regions of validation: we included observations from SW Greenland (Williamson et al. 2018) and NW Greenland (Tedstone et al. 2020, which contains data from both S6 and Upernavik) in our original submission.

2. Concerning the assertion of only using "our own studies": we also included the "independent" dataset of Stibal et al. (2017) in our original submission.
3. Concerning the suggestion that independent studies, i.e. without us as authors, are somehow better: we have studied Greenland glacier algal blooms for many years. Arguably, given our detailed understanding of how our own field datasets were collected, these comparisons are particularly relevant given that comparisons to *"other independent studies"* necessitates utilising field datasets which often lack key meta-data and/or information on the spatial organisation of sampling strategies – as discussed in our first submission.

Nonetheless we have expanded our validation approach as follows:

1. **In the main text, a new time-series comparison to Stibal et al. (2017)'s S6 dataset which complements our existing use of this dataset;**
2. **In the new supplementary, a detailed discussion and comparison to the remote sensing products of Wang et al. (2018) across 4 regions of the GrIS 'dark zone' during summers 2016 and 2017;**
3. **We have identified a limited number of additional observations which we now include in our main validation, though we note that all have very low replication (Fig. 5A):**
    a. **From Stibal et al. (2015/2017) from various locations around the GrIS acquired during 2013 – we only found these counts while re-reading the Wang et al. (2018) manuscript in view of the preceding point, so this was an omission on our side;**
    b. **The datasets of Halbach et al (2022) for S.E. Greenland and Onuma et al. (2022) for Qanaaq ice cap**
4. **See also Supplementary Table 1, which identifies other studies which we are aware of but that we explicitly exclude for reasons that are justified there.**

Concerning the 'bright' margins of the SW GrIS in satellite imagery, **please see responses above and our revised Methods section 'Model validation against observations'.**

L146–149: To support the validity of the model, it would be beneficial to focus on the southwestern region of the GrIS and include a figure comparing the modeled biomass distribution with satellite observations. If comparison with satellite-derived biomass is not feasible, at least a qualitative comparison with satellite imagery should be considered.

While darkness does not equal algal abundance, (see earlier comments), we point the reviewer to Figure 6 in Williamson et al. 2020, which shows the R2 = 0.75 linear relationships between GA_BLOOM outputs and MODIS albedo at K-transect sites S6, S7, S8, S9, S10 for model outputs generated across the entire 2016 melt season. **We have added discussion of the previous validation of GA_BLOOM in SW Greenland into the revised manuscript.**

Concerning the wider opportunities for 'validation' with remote sensing, please see our new Supplementary Note 1.

L178–179: Reference 18 pertains specifically to the southwestern GrIS; this should be clearly stated in the manuscript.

Added as requested

L189: Although Pmax is described in the Methods section, a brief explanation here would be helpful for readers' understanding.

Added as requested

L202: "Results and discussion" seems to lack sufficient discussion regarding improvements in field observations.

Yes, we agree, **this is now fixed with our manuscript reorganisation.**

L206–208: The potential for glacier algal blooms to develop in the inland of the GrIS may also depend on the presence or absence of algal spores in those regions. A brief discussion of the possible origin and dispersal of glacier algae would strengthen the manuscript.

See also our response to Reviewer 1 on much the same topic. In summary: we agree and have **added discussion of this to the introduction**, noting our earlier work that showed the presence of glacier algae (based on omics techniques – Lutz et al. 2018) in sites that have not yet experienced melt, as well as a wider discussion on potential dispersal of cells.

L218: Please clearly state which algal species are represented by the model.

Now added in the revised introduction.

Equation 1: A concise explanation of the constants (2937, 796, –0.000232) should be provided. If these constants were empirically derived from observations in southwestern Greenland, that should be explicitly mentioned. It would also be helpful to define the variable t within this section.

Yes, these are empirically derived from logistic fits to previously published productivity incubation datasets as noted in the original manuscript. **We have added more info for clarity here. T is also defined now.**

Equation 3: The rationale for selecting these particular environmental parameters to determine the growth flag should be clarified.

Please see earlier responses to comments and our **revised Introduction**, which gives the rationale based on existing literature on first-order controls of bloom phenology.

Equation 3: It may be more appropriate to use ice surface temperature (or skin temperature) instead of air temperature. If air temperature is intended to serve as a proxy for melt conditions, skin temperature would provide a more physically consistent indicator. Could outputs from the MAR model be used for this purpose?

While we agree that this could technically be the optimal solution, we strongly suspect that making such a change would have no discernible first or second-order impact on our results

here, for the reason that we already explore the near-surface air temperature threshold parameter space for growth from 0 to 1 degrees C. As MAR energy exchanges are fully coupled between the atmosphere and subsurface and so near-surface air temperatures can only be sustained when the surface temperature ≈ 0 (e.g. Fettweis et al., 2017, The Cryosphere 11:1015-1033), then any discrepancy between near-surface vs surface temperatures is implicitly captured within our investigated parameter space.

We also acknowledge that a practical reason for this choice is that hourly near-surface air temperature outputs for the entire study period were already available, whereas hourly skin temperatures were not. As standard, MAR outputs are at daily resolution, with selected variables such as near-surface air temperature sometimes also output at hourly resolution. Hourly skin temperatures would only be possible with a dedicated re-run of MAR for us over the entire time period, which does not seem like the best use of energy-intensive compute resource.

Finally, we reflect more broadly that it is well established that air temperatures are an excellent proxy for identifying ice surface melt conditions. This is most clear with the example of positive degree day (PDD) modelling, which uses near-surface positive air temperatures in conjunction with empirical degree day factors to calculate snow and ice surface melt (e.g. Hock 2005, Progress in Physical Geography 29:362).

Sensitivity analysis to phenological and environmental parameters: This section is important for understanding the model's dynamics and limitations. I recommend summarizing the key findings of the sensitivity experiments more concisely and incorporating them into the main text.

See earlier responses re. **reorganisation of our manuscript.**

L354–358: Please clarify the rationale for determining the range of Θ values used in the analysis.

This is presented in preceding paragraphs (original manuscript line 302 onwards) that discusses outcomes versus field observations across the range of Θ values included in sensitivity analyses (0 – 50%), whereby lowest and highest Θ give unrealistic growth trajectories, i.e. too extremely too high (0 - 2%), or considerably lower (50%) growth, than observed in-situ for known sites/time periods. Given the large impact of Θ on modelled outcomes, we adopt a wide triangular distribution across 5 – 15% in our QMC ensemble approach.

L358–360: Since the initial biomass of glacier algae at the onset of the bloom season likely varies spatially and interannually, a brief discussion of this point should be added.

We have added this as requested, We re-iterate as per our sensitivity analyses that this has very little impact on modelled bloom outcomes.

L386–398: Because this portion presents validation results and discussion, it would be more appropriate to relocate it to the Results and Discussion section.

Reorganised (see earlier responses).

Figure 6: Please clarify what the differences in marker color intensity represent.

Apologies, our legend got clipped from this figure – corrected now.

Figure 7: Axis labels should be added for both axes, and the ranges of the x- and y-axes should be consistent, as they obscure the apparent underestimation of biomass by the model. The blue solid line and shaded area may be unnecessary. The current figure could mislead readers into thinking that the model reproduces the temporal variability of observed biomass.

Our apologies, the axes labels got clipped – **now fixed.** We include the blue 1:1 line to highlight discrepancies between modelled versus measured biomass.

The reviewer notes "apparent underestimation of biomass by the model". As we note in the manuscript, GA_BLOOM is designed to provide spatially-averaged outputs on glacier algal biomass. Thus, its outputs will be inherently lower than the point observations which dominate the available datasets available for validation, particularly if those observations targeted 'high-biomass ice' (**see revised results/discussion**).

The only study to employ a spatially representative sampling strategy was Tedstone et al. 2020. We acquired n=75 ice samples at randomly generated positions across a 250x250 m sampling area. The resulting spatially averaged abundance estimate shows almost perfect alignment with our model outputs (yellow dot, Figure 7), for an area of Greenland and year on which GA_BLOOM was not developed. This indicates that GA_BLOOM can recreate glacier algal abundance for time periods and regions outside of its original domain.

Re '*the current figure could mislead readers into thinking that the model reproduces the temporal variability of observed biomass.'* it isn't clear how the reviewer feels this could be the case. We would welcome a little expansion to help us address this comment, but note that we **now also make a temporal comparison to Stibal et al. (2017) (Fig. 5B) and, secondarily, a temporal comparison to Wang et al. (2018) (Supplementary Note 1).**

Figure 7: The conditions of the comparison between observed and modeled biomass should be carefully explained in the text. For example, was GA_BLOOM driven by meteorological data from each observation site? If so, how were those data obtained? Were the model results compared using outputs from the same days as the biomass observations?

We have added more information here as requested. In summary, GA_BLOOM was forced using hourly outputs of MARv3.13. We compare measured blooms with modelled blooms by matching the modelled day to the observed day and examining the MAR 10 km pixel which overlaps the observation site.

L463: Feng "S" et al.

Amended

L478: add et al.

Amended

Many thanks again for your detailed review which we believe has helped to improve our paper.

EDITOR COMMENTS

Your manuscript titled "Ubiquitous and expanding glacier algal blooms modelled around the Greenland Ice Sheet" has now been seen by 3 reviewers, and we include their comments at the end of this message. They find your work of interest, but some important points are raised. We are interested in the possibility of publishing your study in Communications Earth & Environment, but would like to consider your responses to these concerns and assess a revised manuscript before we make a final decision on publication.

In particular, while reviewers 1 and 2 provided positive evaluations, reviewer 3 continues to raise substantive concerns regarding the validity of your methodology and the robustness of your conclusions. Therefore, we ask you to either compellingly demonstrate the validity of your model for the whole Greenland Ice Sheet, or soften and caveat your interpretations and conclusions to account for the inherent limitations described by reviewer 3.

We therefore invite you to revise and resubmit your manuscript, along with a point-by-point response that takes into account the points raised. Please highlight all changes in the manuscript text file.

Dear editor,
Many thanks for the second round of comments. We are very pleased to see that reviewers 1 and 2 are now happy with the submission. We have responded to each of reviewer 3's comments below. Based on these **we have now added more details to our methodology re. an abstract description of our modelling approach, added further clarifications on several aspects as requested by Reviewer 3 and updated Supplementary Figure 3 and our comparisons to the remote-sensing dataset of Wang et al. (2018).**

Concerning your request to "either compellingly demonstrate the validity of your model for the whole Greenland Ice Sheet, or soften and caveat your interpretations and conclusions to account for the inherent limitations described by reviewer 3": we believe that our responses to Reviewer 3 should be adequate to support our Greenland wide modelling approach, which is already supported by the other reviewers. Having read the manuscript in detail again, we also believe that it is generally presented in as open and objective a form as possible. For example, we draw your attention to the wording in our conclusions section that already states, e.g.

- "Our findings extend current knowledge on the *potential* distribution of glacier algal blooms around Greenland"
- "Sensitivity analyses reveal that uncertainties in key phenological and environmental model parameters yield considerable spread in our model ensembles…."
- "Weak trends of increasing bloom area through time….*tentatively indicate* that blooms will expand further into the interior of the ice sheet…"

Thus, whilst we have made some edits to our discussion to further emphasise the nature of our modelled outputs (e.g. L317, L326, L369, L374, L395), we believe our work in general is already written in a caveated and nuanced style.

Best wishes,

Chris and Andrew.

Reviewer #1 (Remarks to the Author):

The revised has addressed most of my criticisms and the restructuring/adding the sensitivity analysis/validation section makes a lot of sense.

Many thanks.

The introduction has become too long though (almost 4 pages) -- I understand the authors wanted to address all the reviewers' comments but maybe some of that may have stayed in the rebuttal letter?

We agree in principle. However, we note that Reviewer 3 has now asked for even more information to be included, which consistent with this comment, we push back on. The Editor has not commented on the length of the Introduction. This gives us little room to argue for reducing the Introduction's scope, though we would be happy to discuss this with the editor if deemed necessary. **We have done a little word smithing of longer paragraphs to try and reduce the length slightly.**

The main model equation (Eq. 1, line 377) has remained in the numerical form despite the authors promising an abstract form in the response (based on my previous comment) -- could this be fixed?

Apologies, perhaps we did not understand the request. **We have added a more abstract description of the model both in the text (L387 onwards) and in the equation form of the underlying productivity relationship (L395 onwards) in the revised manuscript –** hopefully this addresses your request?

There is a typo at line 99 (should be "no ice-sheet-wide").

Amended, thanks.

Reviewer #2 (Remarks to the Author):

The responses to reviewers remarks is very convincing and the improvements (structure, validation, discussion/figure about the parameter sensitivities, ...) in the revised version are fine for me.

Thank you for the second positive review.

Reviewer #3 (Remarks to the Author):

Ubiquitous and expanding glacier algal blooms modelled around the Greenland Ice Sheet

Thank you for revising the manuscript. I appreciate the authors' efforts in making substantial changes to address the review comments, and the quality of the paper has improved. However, I found the authors' responses to my comments to be insufficient. Although the authors provided explanations for why certain points were not addressed, I did not find them very convincing. Also, perhaps because the manuscript is now easier to read, I noticed some issues that I had not recognized before. I feel reluctant to raise many additional points again given the considerable effort the authors have already put into revising the manuscript, but please understand that there are still many issues that need to be addressed. I hope the authors will further improve the manuscript by referring to the points below.

Major comments:
1. In my comments on the previous version of the manuscript, I suggested comparing the time series of observed and modeled biomass at several sites across the Greenland Ice Sheet. It is disappointing that in the revised manuscript this comparison is conducted only for the southwestern part of the Greenland Ice Sheet. The authors cite the small number of samples and the lack of spatial representativeness of the observed biomass as reasons for not performing the comparison elsewhere. If so, then doesn't that mean the validity of applying GA_BLOOM to the entire Greenland Ice Sheet cannot be demonstrated? The authors present numerical values comparing observed and modeled biomass outside the southwest, but without a time-series comparison, the validity of the growth model cannot be assessed for the whole Greenland Ice Sheet. In the Appendix, the authors compare satellite-observed and modeled biomass in a time-series manner, but this is also limited to the southwest. While I respect the authors' efforts, shouldn't this paper ultimately restrict the simulation domain to the southwestern region? My conclusion here is also related to what I wrote in Major Comment 2.

We are pleased to note that Reviewer 1 now indicates that *"The revised has addressed most of my criticisms and the restructuring/adding the sensitivity analysis/validation section makes a lot of sense";* they (along with Reviewer 2) are satisfied with the validation that we undertook and have no further comments.

We emphasise that for those quasi-time series measurements available (including completely independent measurements collected by Stibal et al), our model shows consistent temporal trends and averaged absolute values of measured and modelled biomass (Fig. 5b). Likewise, we perform comparison to the available time series satellite datasets across multiple sites, which are only available for the southwestern ice sheet. We could not find any other such datasets in the literature. We reiterate that the comparisons in Fig 5a are to unique location/day combinations of field datasets.

The statement above that we "*cite the small number of samples and lack of spatial representativeness….as reasons for not performing the comparison elsewhere"* is incorrect. We use all available datasets Greenland-wide in our model validation. We exclude only a very small number of samples, exclusively when the original study explicitly states they were not collected in a spatially representative manner (see Supplementary Table 1 where this was clearly detailed in our last submission). We would be delighted to perform comparisons elsewhere but the ground data are not available.

Zooming out, glacier algal bloom propagation is known to be consistent not only around the Greenland Ice Sheet (i.e. field knowledge from south, southwest, mid-west, northwest and east Greenland – all datasets included in our validation) but also across the wider

cryosphere. We have studied blooms in multiple locations around Greenland, and in Svalbard, Arctic Sweden, the European Alps, Patagonia, and Signy Island, Antarctica, and have consistently observed the same bloom dynamics across locations.

Finally, we again emphasise that applying an empirically derived growth-environment relationship across a larger domain is a very common strategy. For example, this is the basis of much marine phytoplankton and biogeochemical modelling, where laboratory- or region-derived relationships are propagated over ocean-basin to global scales using spatially explicit environmental forcing. Our model follows this established approach. We use a model developed directly for GrIS glacier algal blooms, explore its sensitivity, conduct a simulation ensemble, and compare against all available glacier algal abundance datasets Greenland-wide.

2. Looking at Eq. (1), if P(0) is set to 1 ng DW ml^-1, then Gp(0) becomes approximately 796 ng DW ml^-1, implying a 796-fold increase within one hour. Can such a growth rate realistically occur? According to the description in L57, the doubling time is 3.75–5.5 days, which is far from this estimate. With this equation, the growth rate decreases once algal biomass reaches a certain level, so the model may appear to behave realistically in that regime. However, doesn't the formulation have a structural problem in that it allows algae to increase extremely rapidly where there are almost no algal cells present? This behavior can also be seen in Fig. 1. This may be because the model is an empirical equation derived from observations in the dark zone of the southwestern Greenland Ice Sheet, where glacier algae are abundant. Applying this model within the dark zone where glacier algae are already prevalent may be acceptable, but shouldn't we be more cautious about applying it to the entire ice sheet, including inland areas of Greenland where glacier algae are scarce?

We agree that the lower end of the relationship (i.e. 1 ng DW ml) would produce unrealistically high "growth rates", but the application of this relationship is misinterpreted by the reviewer.

First, Eq(1) describes potential *daily* biomass production, not hourly production, so the example given corresponds to a one-day rather than a one-hour increment. Second, we agree that extrapolating the fitted relationship to vanishingly small biomasses would imply unrealistically rapid proportional increases. However, the model is not initialized with such values. As outlined in our manuscript, following a dedicated sensitivity analysis, all simulations are seeded with an initial biomass of 179 ng DW ml^{-1}, rather than 1 ng DW ml^{-1}. At this seed biomass, the unconstrained productivity relationship implies a doubling time (T_d = ln(2) / μ, where μ = ln(biomass_final / biomass_initial)) of 0.4 d under the hypothetical case that all 24 hours are growth-permissive and no losses occur. This increases progressively with biomass as the relationship saturates, e.g. ~1.6 d at 1700 ng DW ml^{-1}, ~2.0 d at 2500 ng DW ml^{-1}, and ~3.9 d at 10,000 ng DW ml^{-1}.

Importantly, these values are not the final modelled bloom growth rates. In the full model, realised daily biomass increase is reduced relative to this relationship because potential production is then multiplied by the fraction of the day for which environmental conditions are suitable for growth, and a population loss term is further applied. Thus, actual modelled growth within surface ice is consistently lower than would be inferred from Eq (1) alone.

As shown by our sensitivity analyses, varying our seed population size over orders of magnitude has little influence on seasonal Pmax or the cumulative biomass production over the melt season. Consistent with this, our model does not generate major blooms in inland regions where favourable growth conditions are only short-lived. Indeed, we draw particular attention to this aspect of our model in the manuscript, whereby we illustrate with reference to Figure 8 that any inter-annual trend in algal biomass through time effectively disappears if

we only consider locations in the domain with an ‘arbitrarily higher, but still smaller Pmax > 2000 ng dw ml’.

Finally, please see our comments above and in our previous round of responses on the common approach of applying growth-environment relationships across larger domains.

3. It would also be better to discuss how the presence of cyanobacteria (cryoconite) and other algal taxa may affect glacier algal biomass. I would like the authors to add a new section and carefully discuss the limitations and outstanding issues of the current model, including the points above. In my previous review, I commented on why the manuscript did not mention cryoconite, which is one of the factors that darken glacier surfaces. The authors responded that they did not address cryoconite because their model does not calculate albedo. However, as the authors also note, glacier algal growth also contributes to surface darkening, so the biological drivers of darkening on the Greenland Ice Sheet likely include both cryoconite and glacier algae. In other words, isn’t it common for the two to co-occur on glacier surfaces? Given that real glacier environments contain glacier algae as well as cryoconite and other algae, might differences in community structure in the environment influence glacier algal growth? In addition, in L72 the authors review that parasitism by chytrids can affect glacier algae, so shouldn’t the manuscript also discuss how such organisms may influence glacier algal growth?

We disagree and note that we agree with Reviewer 1’s comment that the manuscript is already very long given the extra info added in response to the previous round of reviews. On the Greenland Ice Sheet, Cyanobacteria dominate cryoconite holes, glacier algae dominate the overlying surface ice environment. This is generally consistent across most of the glacial cryosphere, whereby these two habitats represent distinct ecological niches dominated by two different assemblages. Only in very few cases (e.g. Tibetan glaciers) do authors report cyanobacterial communities dominating the surface ice – but this is an exception to the norm. With this paper, we are modelling surface ice glacier algal blooms, not cryoconite hole associated cyanobacterial communities. If we were producing an ecosystem-based model for the supraglacial environment then cryoconite holes would be relevant, but we respectfully disagree that cryoconite is important here.

We also reiterate that our focus is glacier algal bloom modelling, not *“surface darkening”*. If the paper were focussed on albedo and darkening, we would agree that it should include discussion of all factors that contribute to darkening (ice physics and weathering crust evolution, abiotic light absorbing particles [e.g. black and brown carbon, other light absorbing minerals], and biological albedo reducers). Glacier algae are one of the many factors that contribute to darkening, but we reaffirm that our paper is not albedo/darkening focussed and thus cryoconite and their associated cyanobacterial populations are out of scope here.

Specific comments:
L25: What does “inter-annual variability in melt” refer to—variability in the amount of melt, or in the duration of the melt season?

Both – **we have added ‘…magnitude and duration’ accordingly into Line 23.**

L27: As the authors themselves recognize, the current model still contains many uncertainties. Therefore, it would be better to avoid presenting such specific numerical values in the abstract.

Happy to remove it if the editor wishes – though we feel it is better to highlight the magnitude of the prediction.

L35: The model expresses biomass in units of “ng DW mL^-1”, but you use “cells mL^-1” here. Could the authors use “cells mL^-1” throughout the paper? Although the conversion is described, “ng DW mL^-1” is not a commonly used unit for comparison with related studies and is not reader-friendly.

As noted, the conversion is included in the paper and ng DW ml^{-1} and cells ml^{-1} are broadly analogous.

L48–49: It seems reasonable—and has been reported in many studies—that when darkly pigmented algae such as Ancylonema bloom, glacier surfaces become darker. However, the claim here appears to differ substantially from previous understanding. Could the authors also add an explanation for why abundant algal cells can be observed even on bright ice? For reference, a recent paper (Traversa et al., 2025) reported a clear correlation between observed Ancylonema biomass and reflectance ratios from satellite and in situ observations. The statement in L48–49 does not seem to describe a universal phenomenon applicable everywhere on the Greenland Ice Sheet.

We think that there is some misunderstanding here. It is not that the ice surfaces are not dark when they are populated by blooms, it is that the satellites return overall brighter images from these more heavily crevassed and hummocky areas of the ice sheet margin. Here the physical surface is topographically far more heterogeneous than, for example, in the “dark zone”. We have been and directly sampled these regions and confirmed abundant glacier algal biomass (in dark ice) (Williamson et al 2020). More detailed information can be found in our responses to the first round of reviews.

L75–78: Even if algal biomass increased during periods of heavy precipitation, that does not necessarily prove that precipitation has no effect on flushing algal cells away. For example, it is possible that some cells were transported downstream while new cells were supplied from upstream. Since glacier algae grow on bare ice surfaces, it seems self-evident that intense rainfall or meltwater would make cells more likely to be washed away.

As per our previous responses, we do not disagree but for the reasons stated previously, this is likely to operate in a dose-dependent manner. Glacier algae are adapted to grow within a constantly melting ice surface and are able to retain themselves within this matrix such that biomass accumulates through time and blooms consistently form. Hence rainfall/meltwater is not a simple flushing mechanism. Cell mobilisation is likely dependent on the impact of rain on the weathering crust state – this is what will ultimately control glacier algal cell liberation or not. Extreme rain can strip back the weathering crust and turn the ice surface into an ice rink, under these situations heavy redistribution of cells is expected (see e.g. Tedstone et al. 2020). Moderate rain can ‘fill in’ the weathering crust, freezing on contact with the ice, trapping cells within the matrix (not flushing them). As the surface energy balance shifts back to promoting weathering crust development (interstitial melt), the cells are ‘unlocked’ and continue growing etc as they were previously (personal observation at site S6 in 2024).

L95–96: This seems to state the opposite of what is written in L48–49. I think readers will be less confused if the manuscript focuses only on more generally applicable facts.

See comment above – glacier algal blooms in the ice marginal area are not reliably constrained by remote sensing. Darkening studies show (As expected) more algae = darker ice. **We have added brackets around the remote sensing part of L49.**

L370: I believe I commented on this before, but which algal species does this model assume for growth? The main glacier algal species include Ancylonema nordenskioldii and

Ancylonema alaskanum, and both grow on the Greenland Ice Sheet. Please clarify in the Methods whether Eq. (1) is intended to calculate the biomass of one of these species, or the total biomass of glacier algae.

This is a full assemblage model – as per previous comments, the introduction now introduces the main species present in Greenland, second paragraph. **We have added clarification in the methods (L 388).**

L156–169: What factors can cause Θ to vary? For example, wouldn't chytrid activity—reviewed by the authors—also affect Θ? Such possibilities should be included in the discussion.

We already discuss this in detail in the Introduction and we do not think this needs revisiting in the Results/Discussion.

Establishing a Quasi-Monte Carlo ensemble approach: It seems that an explanation of the quasi–Monte Carlo ensemble approach is missing from the Methods section. Also, I think most of L202–211 could be moved to the Methods section.

As per previous comments from this reviewer, we moved this section earlier so that our overall modelling approach is clear prior to the major results. This paragraph provides the important context to the modelling approach and definition of triangular distributions.

Monte-Carlo ensembles are a well-established generic method. As we note in the main text, the benefit of the quasi Monte-Carlo approach specifically is in using low-discrepancy sequences rather than pseudorandom numbers, which allows to reduce variance and sample the full space using many fewer model runs that we would need with regular Monte-Carlo sampling. Per our code available in the repository, we use the popular/standard code implemented in the Python package SciPy (docs.scipy.org/doc/scipy/reference/generated/scipy.stats.qmc.Sobol.html).

L208–211: One point to note is that the logic in these lines would apply only if GA_BLOOM is an equation that can be applied across the entire Greenland Ice Sheet. The authors should first demonstrate that the modeled temporal evolution of biomass is applicable to other regions of the Greenland Ice Sheet as well. I am not convinced that Eq. (1), which was empirically derived from observations in southwestern Greenland, can be applied to the Greenland Ice Sheet as a whole, where multiple species co-exist. Shouldn't the algal growth process itself be carefully validated before focusing on tuning model parameters?

Please see earlier comments. Multi-species coexistence is not relevant here – glacier algal assemblages are highly consistent across all sampled localities around Greenland (and indeed much of the wider northern hemisphere; only in the southern hemisphere have we identified novel glacier algal phenotypes and cryptic diversity). As also noted previously, it is a common modelling approach to apply empirically derived growth-environment relationships across larger domains. Our approach follows this established modelling logic, whilst explicitly acknowledging the limitations in current modelling capabilities and emphasising that future measurements from additional regions of the ice sheet are needed.

L298–301: Looking at Feng et al. (2022), the occurrence frequency of dark ice in the terminal area of southwestern Greenland appears to be relatively low, and the model results (Fig. 7A) do not seem to agree with the satellite observations. In addition, as I mentioned previously, darkening in southwestern Greenland has also been reported to be influenced by cryoconite, so shouldn't this be discussed more carefully?

Please see earlier comments on the difference between remote sensing and ground measurements in the most ice marginal zones and on the topic of cryoconite. It is also interesting to note here that studies such as Feng et al. (2022) often report on ‘dark ice’ dynamics across different sectors of the ice sheet, ultimately invoking glacier algae as the dominant darkening agent and typically do not themselves focus discussion on cryoconite.

L325–347: Given that validation across the entire Greenland Ice Sheet is insufficient and the simulations rely on a simple set of assumptions, do the numerical values presented in this subsection really have meaningful significance? I suggest deleting this subsection. At the very least, it would be better to restrict the analysis domain to southwestern Greenland when presenting these numbers.

We respectfully note that across the three reviewers, it is reviewer 3 alone who has taken the stance that our validation across Greenland is *“insufficient”*. We identify no reason not to estimate the potential carbon accumulation relative to modelled glacier algal abundance. Furthermore, we have done this across a range of scenarios in potential cellular carbon quotients (estimated directly, using allometric or linear biovolume scaling); i.e. in an as open a manner as possible and trying where possible to communicate any spread in estimates.

L354: Improving the glacier algal growth process assumed by the model is also necessary. The manuscript argues—perhaps more than necessary—for improving in situ observational data, but is that truly the main conclusion of this paper? I hesitate to say this, but it felt as though the authors were attributing the inability to validate the model, and the mismatch between the model and observations, to the sampling methods of the in-situ data.

One important conclusion of this paper is indeed that current ground truth datasets around Greenland are insufficient, in at least two or three respects: (1) spatial representativity at the bloom scale (as opposed to, for example, preferentially sampling only ‘high biomass ice’ at the centimetres scale), (2) samples from areas of the ice sheet not previously sampled; (3) dedicated time-series at other sites. We need more and better sampling of other regions to aid in future modelling and remote sensing efforts. We have recently launched a global community sampling campaign to this end, with first sampling expected during summer 2026.

Our other major conclusions include new knowledge on the least-known aspects of bloom ecology that we need to address in order to improve future modelling efforts; on the potential spatiotemporal distribution of glacier algal blooms around Greenland, highlighting new regions that are priority for future sampling; and a potential increasing trend in bloom extent through time, which may have implications for Greenland’s SMB. Thus, our conclusion related to rigorous observational datasets is one of a series of important outputs of this work.

L372: Could you change the unit from “ng DW mL^-1” to “cells mL^-1”? Many related studies express concentration as cell counts (cells mL^-1). Since I have nothing to compare against, I cannot judge how reasonable the biomass values computed by this model are. In particular, I would like to know what the initial value of 178 ng DW mL^-1 corresponds to in cells mL^-1.

Please see earlier comment. 179 ng DW ml is 212 cells ml. In general, divide by 0.84 to get to wet weight units.

L381: Is my understanding correct that H(t) can reach a maximum of 3 if the conditions for each component are satisfied? Please add a clarification.

Thanks for spotting this. In the equation we had omitted to divide the sum by 24 (hours) to yield the fraction of the day which is productive, i.e a value which ranges

between 0 and 1. This is now fixed and, we believe, clear enough. We have also added enclosing brackets around all three conditions.

We take this opportunity to clarify to the reviewer that the symbol '&' is the binary AND operator per widespread usage. Thus,

1 & 1 & 1 = 1,

while

1 & 1 & 0 = 0.

Per the existing methods text, the equation is applied to the hourly data, i.e. each hour of the day is either productive (1) or not productive (0). These values are then summed for the 24 hour period (yielding values 0-24) which are finally divided by 24 to yield the fraction of the day which is productive. The reviewer is also welcome to look at our code if they wish.

L406–412: For the entire Greenland simulation, what value did you use for the initial biomass P(0)? What values did you use for the other parameters, such as the loss rate?

This information is found in the section 'Establishing a Quasi-Monte Carlo ensemble approach'. We located this information here, not in the Methods, because the choice of values reflects the information presented in the preceding section 'Sensitivity analysis to phenological and environmental parameters'.

We have moved the statement of using a fixed P(0)=179 ng dw ml for the entire Greenland simulation from the sensitivity analysis section into the QMC approach section as we concur that this value was difficult to find previously.

In the Methods section 'Description of glacier algae model', we have also added a signpost to the section 'Establishing a Quasi-Monte Carlo ensemble approach' concerning the choices of parameters for the Greenland-wide simulations.

L407: Please specify the names of the MAR physical variables used as inputs to the model. I can guess from Eq. (2), but I'm not certain.

Added as requested. ***"We used the MAR variables TT (near-surface temperature), SWD (shortwave-down radiation), SHSN2 (snowpack height above ice) and MSK (ice mask) as inputs to GA_BLOOM."***

L449–451: I did not understand what this means. Does it mean that you calculated the mean and standard deviation for each observational dataset listed in Table S1 in the Appendix? Since algal biomass varies over time and GA_BLOOM is also a model that simulates the temporal evolution of biomass, is it meaningful to compare using such averaged values? The sampling dates also differ among the observational datasets. Wouldn't it be better to conduct multiple comparisons with time-series observational datasets like Fig. 5b and discuss them carefully, as you did in L260–280?

There is some misunderstanding here. **The answer to this question is already available in at L452-4354 of the previous submission:** "For each unique location/day observation, we compare to the modelled population size on the day in the pixel of the model domain which intersects the observation's location". **I.e. we did *not* undertake any temporal (multi-day) averaging.** We only averaged together samples acquired on the same day per location. See subsequent responses re. time series comparisons as per L260-280.

Figure 5b: The observations from Stibal et al. (2017) are also from southwestern Greenland. Is it not possible to compare the model with time-series biomass observations from other regions?

There are no other time-series biomass observations that we are aware of, only point samples. This likely stems from the complexity and cost of establishing field camps on the ice sheet, in comparison to point sampling which can be achieved by helicopter or fixed-wing aircraft in < 1 day. That said, Fig 5A includes all unique location/day comparisons around Greenland.

Supplementary Figure 3: Could you show where Sites 1–4 are located within southwestern Greenland using summer satellite imagery? In my previous comments, I pointed out that the terminus area in southwestern Greenland is white; please show in a figure whether the model is being validated in such regions. This information would also be useful for readers.

We have inserted a panel to S.Fig. 3 showing the locations as requested. Concerning the 'white' terminus areas: Wang et al 2018 explicitly restricted retrieval of algal blooms to the 'dark ice area' only and so it is impossible to use their dataset to consider bloom dynamics nearest the margins (their Sect. 2.3, 'Accordingly we calculated the ratio index [diagnostic of algae] for each dark ice pixel'). Please see earlier responses related to 'the terminus area in southwestern Greenland is white', where we have confirmed through ground sampling that this is not the case.

Supplementary Figure 3: Since I would like to assess the agreement between the model and observations, could you add the coefficient of determination (R^2) to the figure? Similarly, could you add R^2 to Fig. 5b as well?

We thank the reviewer for this suggestion but argue that the approach does not yield further insight into model performance. For the sake of openness we are happy to share the R2 values here, which range 0.07 – 0.78 across Supplementary Figure 3 (mean = 0.41), and R2 = 0.23 for figure 5b. However, such a linear regression analysis is not very informative here because of the issues with the spatial representativeness of point samples and the mis-match between such 'time series' and GA_BLOOM outputs. We have discussed these issues at length in the manuscript and in previous responses to reviews, but take this opportunity to make some further specific remarks relevant to this comment.

First, as we have noted on several occasions, the low replication of most field studies introduces very large error into spatial averages of glacier algal biomass. We reassert here that much higher replication is needed to adequately capture the true spatial mean of glacier algal blooms; we have quantified this in a paper currently in preparation by sampling n = 200 samples across a glacier algal bloom, showing how lower replications of e.g. n = 10 gives ~ 30 – 50% mean average error from the true spatial mean. Thus, we find that the currently available point sample datasets (excepting that from Tedstone et al. 2020 in UPE) provide information on the order of magnitude of blooms across sites/times rather than reliable exact spatially averaged values to regress against. The retrieval algorithm of Wang et al. (2018) is trained on a subset of these ground truth datasets and the outputs themselves are not provided with any uncertainty bonds, limiting interpretation of the variability in their time series presented in Sup Fig. 3 (already discussed in detail in Supplementary Note 1).

Second, we have already demonstrated in our revised manuscript that GA_BLOOM aims to capture the broader bloom trajectory through time, rather than the high short-term variability that is apparent in ground observations – arguably making it ideal for larger-scale ice-sheet wide simulations. Consistent with this, we have always integrated GA_BLOOM outputs to seasonal metrics per location (Pmax and ∑GN). In this respect we already present our

comparisons to the ‘time series’ of Stibal et al. (2017) by calculating the mean and median trajectories in their observations through time (though we note that the authors themselves did not present their data as such), and integrating these and the contemporaneous GA_BLOOM outputs into PMAX and ∑GN metrics; showing very strong congruence. **We have now also undertaken this comparison for the biomass retrievals of Wang et al. (2018) over the 4 sites and 2 years, again showing good congruence between both datasets. We include these metrics in Supplementary Table 2.**

Dear Nicola,

Many thanks for your tentative acceptance of our manuscript, for the further guidance via email on final submission, and for waiting until now for this submission given our field-work delays.

We have now followed all editorial formatting guidelines and have resubmitted our manuscript as requested.

The sole outstanding editor comment as per our recent emails was:

Would it be feasible to add a brief section summarising the main limitations of your model and explaining how these may influence the Greenland-wide simulations? I think this would be helpful for readers, particularly in providing a concise overview in one place. Otherwise, I would recommend clearly addressing our editorial request in the rebuttal letter, explicitly explaining how this point was considered but not developed further in the manuscript.

To reply to this comment:

We have conducted yet another review of our manuscript (please see previous detailed responses in our last submission) to try and identify any additional places where we could draw even more attention to the current limitations of our (or any) modelling approach. Given that this is one of the fundamental points of our paper, we have not been able to identify any. This includes adding an additional section summarising the main limitations of our model and how they influence our simulations. Essentially, all of the manuscript is on this point and we include several summaries of this throughout, including in our conclusions section, which reads:

… Sensitivity analyses reveal that uncertainties in key phenological and environmental model parameters yield considerable spread in our model ensembles, underscoring the need for improved *in situ* measurements. Top-down controls and physical removal of glacier algal cells from the ice surface remain particularly poorly constrained yet have a large impact on model outcomes. We also emphasise the current dearth of spatially resolved ground-truth datasets on glacier algal abundance that limits larger-scale model calibration and validation, despite the intensity of their study over the past decade…

Thus, we hope our explanation here of how this point was considered but why it was not further developed is clear.

Many thanks and best wishes,

Chris and Andrew.
